# Effect of Financial Incentives on Breastfeeding: A Cluster Randomized Clinical Trial

**DOI:** 10.1001/jamapediatrics.2017.4523

**Published:** 2017-12-11

**Authors:** Clare Relton, Mark Strong, Kate J. Thomas, Barbara Whelan, Stephen J. Walters, Julia Burrows, Elaine Scott, Petter Viksveen, Maxine Johnson, Helen Baston, Julia Fox-Rushby, Nana Anokye, Darren Umney, Mary J. Renfrew

**Affiliations:** 1 School of Health and Related Research, University of Sheffield, Sheffield, England; 2 Faculty of Health Sciences, University of Stavanger, Stavanger, Norway; 3 Faculty of Life Sciences and Medicine, Kings College London, London, England; 4 Health Economics Research Group, Brunel University London, Uxbridge, England; 5 Department of Engineering and Innovation, Open University, Milton Keynes, England; 6 Mother and Infant Research Unit, School of Nursing and Health Sciences, University of Dundee, Dundee, Scotland

## Abstract

**Importance:**

Although breastfeeding has a positive effect on an infant’s health and development, the prevalence is low in many communities. The effect of financial incentives to improve breastfeeding prevalence is unknown.

**Objective:**

To assess the effect of an area-level financial incentive for breastfeeding on breastfeeding prevalence at 6 to 8 weeks post partum.

**Design, Setting, and Participants:**

The Nourishing Start for Health (NOSH) trial, a cluster randomized trial with 6 to 8 weeks follow-up, was conducted between April 1, 2015, and March 31, 2016, in 92 electoral ward areas in England with baseline breastfeeding prevalence at 6 to 8 weeks post partum less than 40%. A total of 10 010 mother-infant dyads resident in the 92 study electoral ward areas where the infant’s estimated or actual birth date fell between February 18, 2015, and February 17, 2016, were included. Areas were randomized to the incentive plus usual care (n = 46) (5398 mother-infant dyads) or to usual care alone (n = 46) (4612 mother-infant dyads).

**Interventions:**

Usual care was delivered by clinicians (mainly midwives, health visitors) in a variety of maternity, neonatal, and infant feeding services, all of which were implementing the UNICEF UK Baby Friendly Initiative standards. Shopping vouchers worth £40 (US$50) were offered to mothers 5 times based on infant age (2 days, 10 days, 6-8 weeks, 3 months, 6 months), conditional on the infant receiving any breast milk.

**Main Outcomes and Measures:**

The primary outcome was electoral ward area-level 6- to 8-week breastfeeding period prevalence, as assessed by clinicians at the routine 6- to 8-week postnatal check visit. Secondary outcomes were area-level period prevalence for breastfeeding initiation and for exclusive breastfeeding at 6 to 8 weeks.

**Results:**

In the intervention (5398 mother-infant dyads) and control (4612 mother-infant dyads) group, the median (interquartile range) percentage of women aged 16 to 44 years was 36.2% (3.0%) and 37.4% (3.6%) years, respectively. After adjusting for baseline breastfeeding prevalence and local government area and weighting to reflect unequal cluster-level breastfeeding prevalence variances, a difference in mean 6- to 8-week breastfeeding prevalence of 5.7 percentage points (37.9% vs 31.7%; 95% CI for adjusted difference, 2.7% to 8.6%; *P* < .001) in favor of the intervention vs usual care was observed. No significant differences were observed for the mean prevalence of breastfeeding initiation (61.9% vs 57.5%; adjusted mean difference, 2.9 percentage points; 95%, CI, −0.4 to 6.2; *P* = .08) or the mean prevalence of exclusive breastfeeding at 6 to 8 weeks (27.0% vs 24.1%; adjusted mean difference, 2.3 percentage points; 95% CI, −0.2 to 4.8; *P* = .07).

**Conclusions and Relevance:**

Financial incentives may improve breastfeeding rates in areas with low baseline prevalence. Offering a financial incentive to women in areas of England with breastfeeding rates below 40% compared with usual care resulted in a modest but statistically significant increase in breastfeeding prevalence at 6 to 8 weeks. This was measured using routinely collected data.

**Trial Registration:**

International Standard Randomized Controlled Trial Registry: ISRCTN44898617.

## Key Points

**Question:**

Does offering financial incentives for breastfeeding increase breastfeeding at 6 to 8 weeks post partum in areas with low (<40%) breastfeeding prevalence?

**Findings:**

In this cluster randomized clinical trial that included 10 010 mother-infant dyads in England, randomization of electoral ward areas to a financial incentive for breastfeeding compared with usual care resulted in a modest but significantly greater prevalence of breastfeeding at 6 to 8 weeks (37.9% vs 31.7%).

**Meaning:**

Financial incentives may improve breastfeeding rates in areas with a low baseline prevalence.

## Teaser

This randomized clinical trial evaluates the use of financial incentives for breastfeeding in community settings with a low baseline prevalence.

## Introduction

Breastfeeding is associated with a positive effect on an infant’s life chances, survival, development, and health, including protection against childhood infections, obesity, and diabetes; nursing women are also protected against breast cancer.^1^ The importance of breastfeeding in promoting health and development is reflected in national and international policy recommendations and guidance.^3^ However, there are considerable long-standing social and cultural barriers to breastfeeding in many settings. Breastfeeding in many countries is sexualized in public discourse and the media, resulting in a powerful disincentive to breastfeed mediated through embarrassment and fear.^5^ Breastfeeding prevalence has been low in many low-income communities in high-income countries for generations. Over the past 25 years, breastfeeding rates in such communities have not risen in response to a range of policy developments,^6^ and no trials of support interventions have been effective in increasing breastfeeding prevalence.^8^

There is increasing interest in the role of financial incentive programs to meet the health needs of children^9^ and financial incentives are increasingly being used to improve maternal and newborn health.^10^ However, evidence as to whether financial incentives are effective in increasing breastfeeding prevalence is weak.^15^ Although incentives that support breastfeeding are being implemented (eg, women in France are given paid breastfeeding breaks during the working day),^11^ incentives that support infant formula are also being implemented (eg, the UK national statutory scheme [Healthy Start] provides vouchers of £6.20 [US$7.75] per week in the first year that can be exchanged for infant formula for women in receipt of welfare payments, many of whom live in areas with low breastfeeding prevalence). The objective of the Nourishing Start for Health (NOSH) cluster randomized trial was to assess the effects of an area-level financial incentive scheme for breastfeeding on breastfeeding prevalence at 6 to 8 weeks postpartum in areas with historically low (<40%) breastfeeding rates at 6 to 8 weeks post partum.

## Methods

### Trial Design

We conducted a cluster randomized clinical trial in electoral ward areas situated in 5 local government areas in the north of England (April 1, 2015, to March 31, 2016). The trial protocol, approved by the National Health Service and local authority Research Governance and Research Ethics Committees, has been published^17^ and is available in the Supplement. This trial randomized clusters (electoral ward areas that are the geographic unit for which routine aggregated data on infant feeding is routinely reported). Thus, consent to take part in the trial was obtained from local government areas and the leads for infant feeding services in these areas. As women opted into the scheme, applications to join were understood to be implicit consent to take part in the research.

### Study Site and Participants

Mother-infant dyads were eligible for the financial incentive if the estimated (or actual) infant birth date fell between February 18, 2015, and February 17, 2016 (hence, the infant would be aged 6 weeks between April 1, 2015, and March 31, 2016), and their mother was 16 years or older and lived within an intervention electoral ward area.

### Randomization

Electoral ward areas (not individuals) were randomly allocated to intervention or control using a 1:1 cluster random allocation sequence with stratification at local government area level (with randomly selected block size of 2 or 4). A statistician (one of us, S.J.W.), who was blinded to ward names, used a computer-generated random sequence allocation method.

### Intervention

Key elements of the financial incentive intervention were developed with local clinicians, commissioners, and communities during the pretrial feasibility study.^18^ Women in the intervention clusters were informed about the scheme and invited to join by clinicians (mainly midwives and health visitors). A web-app–facilitated postal address eligibility checking and a booklet describing the scheme were made available to clinicians and distributed to children’s centers and other public places. The booklet described the benefits of breastfeeding, identified sources of infant feeding support, and described the vouchers as “a way of acknowledging the value of breastfeeding to babies, mums, and society, and the effort involved in breastfeeding.” The booklet informed women that the “NOSH Scheme is being tested by researchers.” Initial uptake of the scheme was slower than in the pretrial feasibility study, as many women had not heard about the plan; therefore, from trial month 4, banner posters were put in hospital waiting rooms, social media (Facebook) advertisements were posted to women in the intervention areas, and from month 6, 4 clinicians were employed part-time to disseminate information to local infant feeding services.

The incentive intervention was offered to women conditional on their infant receiving any breast milk. The scheme offered shopping vouchers worth £40 (US$50) 5 times based on infant age: 2 days, 10 days, 6 to 8 weeks, 3 months, and 6 months (ie, up to £200/US$250 in total). Vouchers were exchangeable at supermarkets and other retail shops with no restriction on allowable purchases. Receipt of vouchers was conditional on mothers signing a form stating that “my baby is receiving breast milk” and a countersignature from a clinician for the statement “I have discussed breastfeeding with mum today.”

Clinicians were asked to notify the research team confidentially if they had a concern that an infant was not receiving breast milk without the claim being jeopardized. Mothers’ mailed claim forms, claims, and verification of clinicians’ signatures were processed independently of the research team. Financial incentives were delivered directly to mothers either as vouchers or prepaid gift cards.

### Usual Care

The incentive scheme was offered in addition to usual care for all women in all areas. Usual care was delivered by midwives, health visitors, and breastfeeding peer supporters working in a variety of maternity, neonatal, and infant feeding services. All hospitals and community services had UNICEF UK Baby Friendly Initiative accreditation and were implementing the UNICEF UK Baby Friendly Initiative standards.

### End Point and Data Collection

The primary end point was routinely collected electoral ward area-level period prevalence of any breastfeeding (ie, exclusive or nonexclusive) at 6 to 8 weeks post partum between April 1, 2015, and March 31, 2016. Area-level 6- to 8-week breastfeeding prevalence is a UK national public health outcome measure.^20^ Two secondary outcomes were included for the same period as the primary outcome: the period prevalence of breastfeeding initiation and exclusive breastfeeding at 6 to 8 weeks. All area-level data were collected routinely (and independently of the trial) by those delivering routine infant feeding services (midwives, health visitors, and primary care physicians) and collated by the local National Health Service Trust, Local Authority, or Child Health Information team. The protocol specified collection of individual-level secondary outcomes to inform a cost-effectiveness analysis (duration of exclusive and any breastfeeding, and the number of consultations with clinicians concerning gastrointestinal infection, otitis media, respiratory tract infections, and atopic eczema), but it was not possible to obtain these data.

### Statistical Analysis

The original sample size calculation^17^ assumed that individual-level mother-infant feeding status outcome data would be collected using a questionnaire; however, in the pretrial feasibility stage, it became clear that this method would lead to poor estimates due to respondent bias. Therefore, routinely collected electoral ward area-level breastfeeding prevalence data were used. The unit of analysis was the electoral ward area, and breastfeeding prevalence was treated as a continuous outcome. A sample size calculation based on a baseline mean (SD) area-level 6- to 8-week prevalence of 28.2% (6.9%), a power of 80%, and a 2-sided significance level of 5% determined that 47 areas per trial group would be required to detect a 4 percentage point difference between intervention and control (this was the smallest effect size that it was feasible to study given resource constraints).

We gained local stakeholder consent to conduct the trial in 170 electoral ward areas (average population, 9500). These sites were situated in 5 adjacent local government areas in the north of England (Bassetlaw, Doncaster, North Derbyshire, Rotherham, and Sheffield). Of these 170 electoral ward areas, 92 had a 6- to 8-week breastfeeding prevalence of less than 40%, based on the most recent area-level breastfeeding data available, and were included in the trial.

For our main analysis of the primary outcome measure, a weighted multiple linear regression model was used to estimate the intervention effect after controlling for baseline breastfeeding prevalence and local government area. Weights were calculated using the method of Donner and Klar^21^ and were based on an intraclass correlation coefficient estimated from the data using the method of Fleiss and Cuzick.^22^

The primary analysis was by intention-to-treat at the electoral ward area (cluster) level. Electoral ward area-level breastfeeding prevalences were calculated on a complete case basis in which the denominator was the number of infants with known breastfeeding status; infants for whom we had missing outcome data were not included in the analysis.

We conducted the following secondary analyses for our primary outcome. First, we calculated the unweighted, unadjusted effect size and tested for significance using an independent samples *t* test. Second, we calculated the effect size using a weighted regression, adjusting for the following baseline cluster-level covariates known to be associated with breastfeeding: Index of Multiple Deprivation,^23^ the proportion of women aged between 16 and 44 years in 2011, the proportion of the population who self-identified as nonwhite in the 2011 UK Census, and the count of births in 2015.

To explore how the effectiveness of the intervention evolved over time as knowledge of the scheme increased, we calculated the effect size using the same regression model as for the primary analysis, but for each quarter of the trial period separately. We tested for a linear increase in effect size over the 4 quarters using a regression of the primary outcome on the interaction between calendar quarter and intervention group, adjusting for local government area and baseline.

For the secondary outcomes of breastfeeding initiation and 6- to 8-week exclusive breastfeeding, we estimated the intervention effect using a weighted linear regression with adjustment for local government area and baseline prevalence. Due to unavailability of electoral ward area-level data on either of the secondary outcomes, we used the baseline 6- to 8-week breastfeeding prevalence as a proxy measure in each case.

Statistical analyses were carried out using SPSS, version 21 (SAS Institute) and R, version 3.4.1 (R Foundation). All tests were 2-sided with a significance threshold of 5%.

## Results

**Figure 1.**
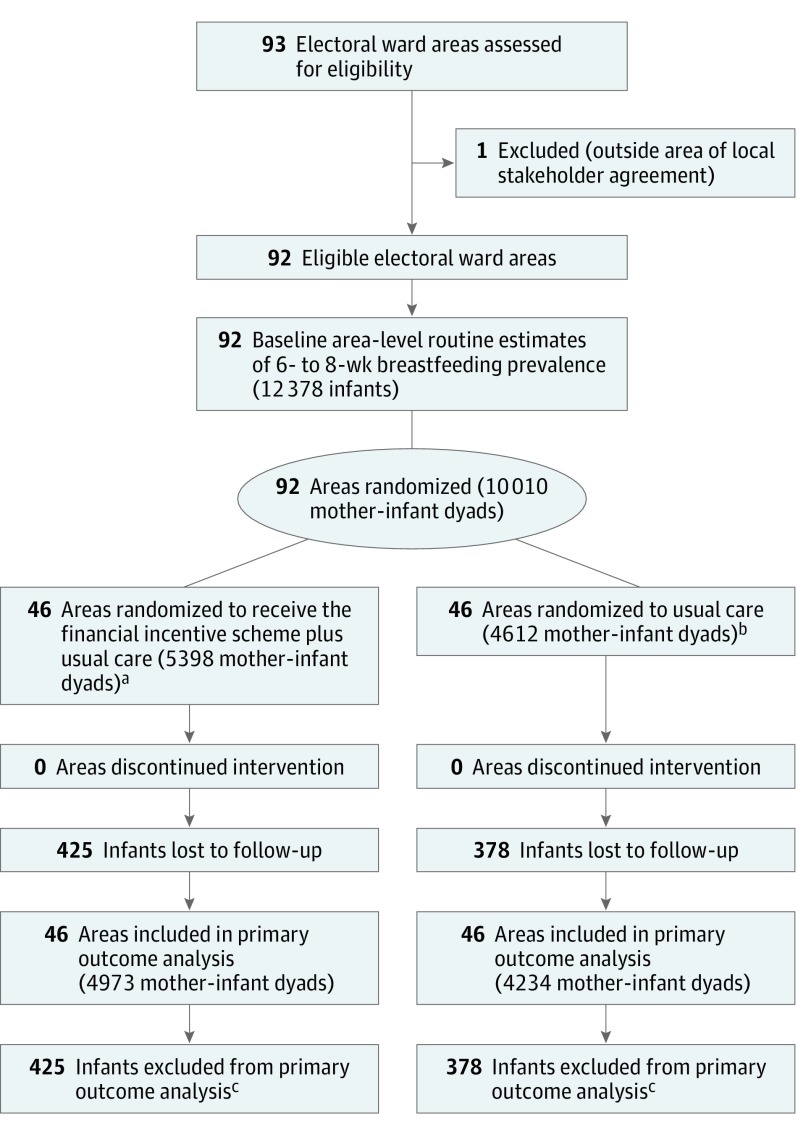
Cluster Recruitment and Follow-up ^a^Mean (SD) cluster size, 117 (78). ^b^Mean (SD) cluster size, 100 (68). ^c^No 6- to 8-week feeding status recorded.

Table 1 describes the baseline characteristics of the 92 intervention and control wards. A flowchart is presented in Figure 1. During the intervention period, 10 010 infants were due for a 6- to 8-week postnatal check (n = 5398 intervention, n = 4612 control). The mean area-level deprivation scores were higher (more deprived) than the mean for England (21.7).

**Table 1.** Baseline Characteristics of Intervention and Control Electoral Ward Areas

Characteristic	Control Group (n = 46)	Intervention Group (n = 46)
Annual No. of infants due a 6- to 8-wk postnatal check, median (IQR)	130 (76-175)	129 (91-180)
Baseline 6- to 8-wk breastfeeding prevalence, mean (SD), %	27.4 (7.3)	28.7 (6.5)
Adult population, median (IQR), No.	8090 (3863-13 342)	11 284 (4532-14 028)
White population, median (IQR), %^a^	97.9 (97.0-98.3)	97.5 (96.0-98.0)
Deprivation score, mean (SD)^b^	28.7 (10.3)	28.0 (9.8)
Women aged 16-44 y, mean (SD), %^a^	37.4 (3.6)	36.2 (3.0)
Total births in the trial period, median (IQR), No.	75.5 (39-145)	101.0 (54-160)

Abbreviation: IQR, interquartile range.

^a^ Derived from the 2011 UK Census.

^b^ Index of Multiple Deprivation 2015. Electoral ward-level scores were population-weighted means of Lower Level Super Output Area–level scores (range, 0.48 to 92.6; England mean, 21.7). Higher score indicates more deprivation.

During the trial, 2496 of 5398 (46.2%) eligible infant-mother dyads registered with the scheme, and claims for vouchers were submitted by mothers for 2179 (40.4%) of all eligible infants (including 25 sets of twins). Voucher claims at 6 to 8 weeks (the time for the trial primary outcome) were made for 1827 (33.8%) of all eligible infants, and by the end of the 6-month scheme mothers had claimed 1 or more vouchers for 2179 (40.4%) of all eligible infants (Table 2).

**Table 2.** Voucher Claims for the 5 Claim Points in 5398 Eligible Infants

Infant Age and Claim Point	Claims for Vouchers, No. (%)
2 d	2169 (40.2)
10 d	2105 (39.0)
6-8 wk	1827 (33.8)
3 mo	1449 (26.8)
6 mo	1022 (18.9)

Almost all claims (8239 [96.2%]) were countersigned by midwives or health visitors; other signatories included nurses, primary care physicians, pediatricians, nursery nurses, breastfeeding support workers, and midwife support workers (528 signatories in total). During the trial, clinicians signing claims forms were asked to report any concerns they had that an infant was not receiving breast milk without the voucher claim being invalidated. It was not known whether the potential to receive an incentive led to inaccurate self-reporting by mothers to their clinicians. To assess the veracity of the claims and the outcome data, information was analyzed from all contacts with clinicians involved in delivering the intervention to 2179 eligible mother-infant dyads who claimed vouchers. This included 42 group meetings, 418 telephone calls with the scheme administrators, and 35 researcher-led qualitative interviews. Clinicians reported 19 cases with which they had some uncertainty as to whether the infant was receiving breast milk.

There were 803 (8.0%) infants for whom no 6- to 8-week infant feeding status was recorded, the majority of whom (762 [94.9%]) were from 1 local government area (Rotherham). The proportion of missing data was 7.9% (425 of 5398) in the intervention group and 8.2% (378 of 4612) in the control group (χ^2^ = 0.31, *P* = .58).

The primary outcome—mean cluster-level 6- to 8-week breastfeeding period prevalence—for April 1, 2015, to March 31, 2016, was 31.7% (95% CI, 29.4-34.0) in control areas and 37.9% (95% CI, 35.0-40.8) in intervention areas (Table 3). The trial resulted in a crude unweighted increase in breastfeeding prevalence of 6.2 percentage points (95% CI, 2.4-10.0; *P* = .002) in favor of the intervention. After adjustment for baseline area-level breastfeeding prevalence and local government area and weighting to reflect unequal electoral ward area-level variances, the difference between intervention and control was 5.7 percentage points (95% CI, 2.7-8.6; *P* < .001) (Table 3). Adjusting for additional area-level covariates known to be associated with breastfeeding prevalence (Index of Multiple Deprivation, the proportion of women aged 16-44 years in 2011, the proportion of the population who identified as nonwhite in the 2011 UK Census, and the count of births in 2015) resulted in a mean difference of 4.5 percentage points (95% CI, 1.5-7.5; *P* = .003) in favor of the intervention group. The intraclass correlation coefficient for the primary outcome, estimated from the trial data, was 0.024.

**Table 3.** Primary Outcome: Mean Electoral Ward Area-Level 6- to 8-Week Breastfeeding Prevalence

Analysis	Mean Area-Level, % (95% CI)		Mean Percentage Point Difference (95% CI)	*P* Value^a^
	Control Group (n = 46)	Intervention Group (n = 46)		
Primary analysis				
6- to 8-wk breastfeeding prevalence^b^	31.7 (29.4 to 34.0)	37.9 (35.0 to 40.8)	6.2 (2.4 to 10.0)	.002
Analysis by quarter				
Quarter 1: Apr-Jun 15	31.4 (27.5 to 35.3)	34.1 (29.7 to 38.4)	2.7 (−3.3 to 8.6)	.38
Quarter 2: Jul-Sep 15	33.3 (28.6 to 38.0)	37.3 (32.4 to 42.3)	4.0 (−2.9 to 10.9)	.25
Quarter 3: Oct-Dec 15	32.1 (26.6 to 37.5)	38.2 (33.8 to 42.6)	6.2 (−1.0 to 13.3)	.09
Quarter 4: Jan-Mar 16	29.3 (24.7 to 33.8)	41.3 (37.1 to 45.5)	12.0 (5.8 to 18.3)	<.001

^a^ Independent-samples *t* test.

^b^ For the primary outcome, only infants whose feeding status was known were included in the denominator for the breastfeeding prevalence calculation.

**Figure 2.**
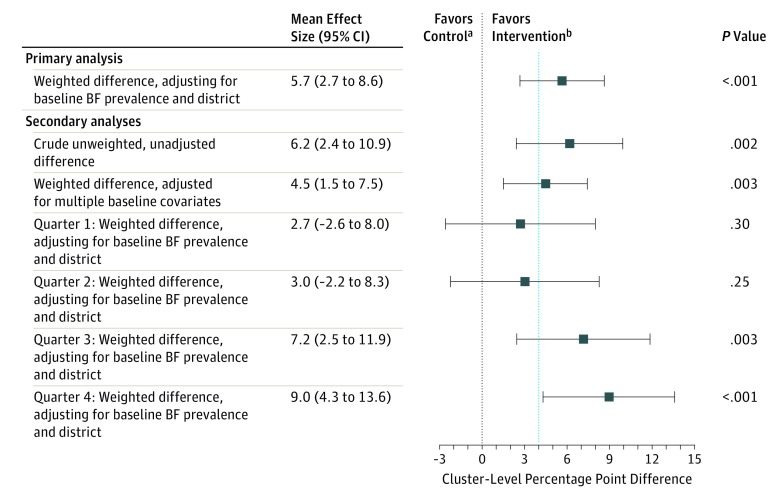
Effect Sizes for the Outcomes Percentage point differences determined as intervention-control. BF indicates breastfeeding. ^a^No effect. ^b^Minimally important difference (4 percentage points).

Figure 2 shows the mean difference in 6- to 8-week breastfeeding prevalence for each quarter, adjusted for local government area and weighted to reflect unequal electoral ward area-level variances. Over time as knowledge of the scheme grew, an increase in effect was seen (*P* = .01 for linear trend), with an effect size in the fourth quarter (January to March 2016) of 8.9 percentage points (95% CI, 4.4-13.5; *P* < .001) in favor of the intervention.

### Secondary Outcomes

For breastfeeding initiation, the mean prevalence was 57.6% (95% CI, 54.1% to 61.0%) in control areas and 61.6% (95% CI, 58.8% to 64.5%) in intervention areas. There was no significant difference between intervention and control groups (mean, 4.1 percentage point difference; 95% CI, −0.4 to 8.6; *P* = .07). After weighting and adjusting for local government area and baseline 6- to 8-week breastfeeding prevalence (as a proxy for the unknown baseline breastfeeding initiation prevalence), there was no significant difference between the intervention and control groups (61.9% vs 57.5%; mean, 2.9 percentage point difference; 95% CI, −0.4 to 6.2; *P* = .08). The intraclass correlation coefficient for breastfeeding initiation prevalence was 0.039.

For exclusive breastfeeding prevalence at 6 to 8 weeks, the mean prevalence was 24.1% (95% CI, 21.8% to 26.4%) in the control areas and 27.0% (95% CI, 24.8% to 29.2%) in the intervention areas. There was no significant difference between intervention and control groups (2.9 percentage point difference; 95% CI, −0.3 to 6.1; *P* = .08). After weighting and adjusting for local government area and baseline 6- to 8-week breastfeeding prevalence (as a proxy for unknown baseline exclusive breastfeeding prevalence), there was no significant difference between intervention and control groups (27.0% vs 24.1%; 2.3 percentage point difference; 95% CI, −0.2 to 4.8; *P* = .07). The intraclass correlation coefficient for exclusive breastfeeding prevalence was 0.018.

## Discussion

Compared with usual care alone, the offer of a financial incentive in addition to usual care resulted in a 5.7 percentage point increase in breastfeeding prevalence at 6 to 8 weeks in areas with low breastfeeding prevalence. Based on a mean baseline prevalence of 28.2%, this represents a relative increase in prevalence of 20.2%. Although there is no consensus as to what constitutes a significant increase in breastfeeding areas with low prevalence, experts in our pretrial consultation thought that any increase would be of value.

To our knowledge, this was the first trial of a financial incentive for breastfeeding offered at a community (area) level. The largest published trial of an intervention to increase breastfeeding (Baby Friendly Hospital Initiative PROBIT trial)^24^ detected a similar effect size at 6 to 8 weeks (6.0%); however, our trial was not conducted in hospitals but in communities with much lower mean 6- to 8-week breastfeeding prevalence (28.2% compared with 85%), and usual care in these communities (and the hospitals in these communities) already included the PROBIT trial intervention (Baby Friendly standards^25^).

Social support and social interventions (eg, financial incentives) can influence health-related behaviors by transforming unhealthy behaviors into healthy behaviors that are witnessed, actively encouraged, and rewarded, and healthy behavior goals are shared.^26^ Because social relationships play key roles in supporting and protecting women who breastfeed,^2^ it was hypothesized that offering the intervention to communities would help to communicate the value of breastfeeding and have a positive influence on those who support women, and thus address some of the complex, financial, organizational, and cultural barriers that limit breastfeeding. Despite financial incentives for breastfeeding being viewed as contentious by some,^27^ almost half (46.2%) of all eligible women joined the scheme.

A recent small trial of financial incentives^28^ that enrolled 36 low-income breastfeeding women in the US Women Infant and Children program verified breastfeeding using direct observation by research staff. Women in our target population lived in communities in which breastfeeding was not the norm and rarely observed in public. To determine the most appropriate and acceptable method for breastfeeding verification for the area-level intervention, the project team engaged in extensive pretrial consultation and feasibility testing with local women, health care providers, public health leads, and service commissioners.^18^ There was no reliable and practical biochemical method of verifying that an infant is breastfed, and strong concerns were voiced that seeking direct proof of breastfeeding (eg, through observation of a feed) would have a negative effect on the relationship between clinicians and women.^18^ We therefore used the method by which infant feeding status is recorded for the purposes of routine data collection in the UK’s National Health Service: a clinician’s assessment based on their interactions with the mother during routine visits at birth and 6 to 8 weeks postpartum (which includes discussions about feeding and may or may not include witnessing the mother breastfeed).

### Limitations

This trial has a number of limitations. First, we used the preexisting country-wide data system that collects information on breastfeeding prevalence at 6 to 8 weeks for public health monitoring purposes that is based on clinician report; however, these reports are not checked for validity. During the trial, mothers in the intervention arm had a financial incentive to report to clinicians that their baby was receiving breast milk and no feasible way was found to verify the truth of these reports. Although clinicians were given the opportunity to report doubts about the veracity of maternal self-report, notes of clinician doubt were rare. This low level may have been because filing a report would require extra paperwork or might in some way jeopardize the clinician-mother relationship. Future studies of financial incentives for breastfeeding may need to develop objective tests (eg, biochemical markers) to provide objective confirmation of breastfeeding. Second, data on area level breastfeeding prevalence were obtainable only for 2 points (initiation and 6-8 weeks). Although data on voucher claims were collected at 5 points (including 3 and 6 months), these data cannot be a proxy for breastfeeding rates, as the numerator excludes breastfed babies for whom claims were not made. Third, without the cost-effectiveness of the trial intervention, it is not possible to determine the full impact of the behavioral and clinical findings for future public health policy. Lastly, as the effect size increased over the 4 quarters of the trial, this suggests that the overall effect on breastfeeding prevalence might have been greater if the trial had tested the intervention over a longer period.

## Conclusions

Financial incentives may improve breastfeeding rates in areas with a low baseline prevalence. Among women in areas of England with breastfeeding rates below 40%, randomization of electoral ward areas to a financial incentive for breastfeeding compared with usual care resulted in a modest but statistically significant increase in breastfeeding prevalence at 6 to 8 weeks. This outcome was measured using routinely collected data. Research is indicated to explore the feasibility of objectively assessing breastfeeding behavior for future financial incentive studies.

## Notes

Supplement. Trial Protocol
