# Supplementary material for: Effect of Financial Incentives on Breastfeeding: A Cluster Randomized Clinical Trial
Source: JAMA Pediatr. 2017 Dec 11;172(2):e174523. doi: 10.1001/jamapediatrics.2017.4523 (PMC5839268; doi:10.1001/jamapediatrics.2017.4523)
Supplement: Supplement. — Trial Protocol [file jamapediatr-172-475-s001.pdf]

Protocol: A randomised controlled trial of a financial incentive (NOSH) to improve breastfeeding in areas with low 6 week breastfeeding rates

REC reference: 13/WM/0299
Trial sponsor: University of Sheffield
Trial sponsor reference: URMS 129897
Trial funder: MRC/ National Prevention Research Initiative (NPRI) Phase 4
Funder reference: MR/J000434/1
ISRCTN NUMBER: 44898617
NIHR CRN Portfolio ID number: 15385
Protocol version: 3.0
Protocol version date: 11.10.14

Role, name and position:	Signature:	Date:
Principal Investigator:<br>Dr Clare Relton<br>Senior Research Fellow		
For the sponsor<br>Kirsty Woodhead<br>Research Ethics Administrator		

Contents

1. Protocol summary
1.1 Summary of trial design
1.2 Trial schema
2. Introduction
2.1 Background
2.1.1 Not breastfeeding (BF) is a major public health problem
2.1.2 Financial incentives for BF
2.1.3 The need for a multi-centre trial
2.1.4 Development and Feasibility stages
2.1.5 Development stage findings
2.1.6 Feasibility stage findings
2.1.7 Changes as a result of the feasibility stage
2.2 The NOSH Trial
2.2.1 Primary objective
2.2.2 Secondary objectives
2.3 Trial activation
3. Selection of clusters
3.1 Sampling frame & recruitment of areas
3.2 Inclusion criteria at the area level
3.3 Areas and clusters identified
3.4 Area initiation & activation
4. Informed consent
4.1. Obtaining informed consent from areas
4.2. Obtaining informed consent from individuals
4.2.1 Consent to participate in the NOSH Scheme
4.2.2 Consent for data collection at the individual level
5. Selection of individual eligible women
5.1. Trial inclusion and exclusion criteria
5.2. Data collection at the individual level
6. Randomisation procedure
7. Trial intervention
7.1 Summary
7.2 Delivery of the intervention
7.2.1 Introduction of the NOSH Scheme into the cluster communities
7.2.2 Induction of healthcare providers
7.2.3 How women join the NOSH Scheme
7.2.4 How women claim NOSH Vouchers.
7.2.5 Type of vouchers
7.3 Verification of breastfeeding
7.3.1 Verification of BF in Quebec 'Nursing Benefit' Scheme
7.3.2 UK routine data collection and verification of BF for outcome measurement
7.3.3 Local stakeholder views on verifying BF
7.3.4 Method of verifying BF in the trial
7.4 Impact on benefits
7.5 Duration of the intervention
7.6 Role of the NOSH Office
8. Data collection
8.1 Units of data collection, and definition of cluster areas
8.2 Primary outcome measure
8.2.1 Definition of BF
8.2.2 Unit of data collection and definition of clusters

8.2.3 Definition of periods of baseline (pre-trial) and during-trial data collection & timetable
8.3 Covariate data collection
8.4 Secondary outcome measures
8.5 Sampling strategy for obtaining mother's self-report data at six months
8.6 Risk to accessing primary outcome data
8.7 Intervention process monitoring
8.7.1 Qualitative data collection
8.7.2 Quantitative data collection
8.8 Methods to protect against sources of bias
9. Statistics & analysis
9.1 Sample size for the NOSH Trial
9.2 Public Health effectiveness
9.3 Economic evaluation
9.4 Mixed methods process/ context evaluation
9.5 Qualitative data analysis
10. Data management guidelines
11. Trial monitoring and oversight
12. Adverse events
13. Risks to delivery of the intervention
13.1 Extensive work with local collaborators
13.2 Continuation sites
13.3 Risks to Sheffield HV involvement in the trial
14. Trial closure
14.1 End of trial
14.2 Archiving of trial documentation
14.3 Early discontinuation of trial
15. Trial management and Trial committees
15.1 Project Management Group
15.2 Trial Steering Committee
16. Ethical and regulatory compliance
17. Sponsorship and indemnity
18. Publication policy
19. Abbreviations
20. References
Appendix 1 Quebec 'Nursing Benefit' Scheme
Appendix 2 Health Visiting Service Specification
Appendix 3 NOSH Booklet
Appendix 4 Protocol version history

1. Protocol summary

1.1 Summary of trial design

**Full title**	A randomised controlled trial of a financial incentive (NOSH) to improve BF in areas with low 6 week BF rates
**Short title/acronym**	The NOSH Trial
**REC number**	REC reference: 13/WM/0299
**Sponsor number and reference**	URMS 129897
**MRC reference**	MR/J000434/1
**ISRCTN number**	44898617
**NIHR CRN Portfolio ID number**	15385
**Design**	A pragmatic cluster randomised controlled trial with: (i) a mixed methods process and context evaluation and (ii) a cost effectiveness evaluation
**Overall aim**	To evaluate the public health impact of the offer of a financial incentive compared to no offer
**Primary endpoints**	Cluster level change in 6-8 week BF rate from baseline
**Secondary endpoints**	Cluster level BF initiation, BF 6-8wk (any & exclusive) Individual baby level duration of BF (any & exclusive), total number of consultations with health professionals, total number of consultations with GPs and hospital admissions concerning GI infections, otitis media, respiratory tract infections, atopic eczema, all other causes
**Target accrual**	68-82 clusters for the RCT + (3 continuation sites)
**Inclusion criteria**	All clusters with mean 6-8 week BF rates of <40%
**Exclusion criteria**	All clusters with mean 6-8 week BF rates of ≥40%
**Planned number of sites**	68-82 clusters for the RCT + (3 continuation sites)
**Treatment summary**	Offer of a financial incentive to breastfeed
**Definition of end of trial**	The end of the trial will be seven months after the end of the birth eligibility period (17th February 2016)

Figure 1.2 Trial schema

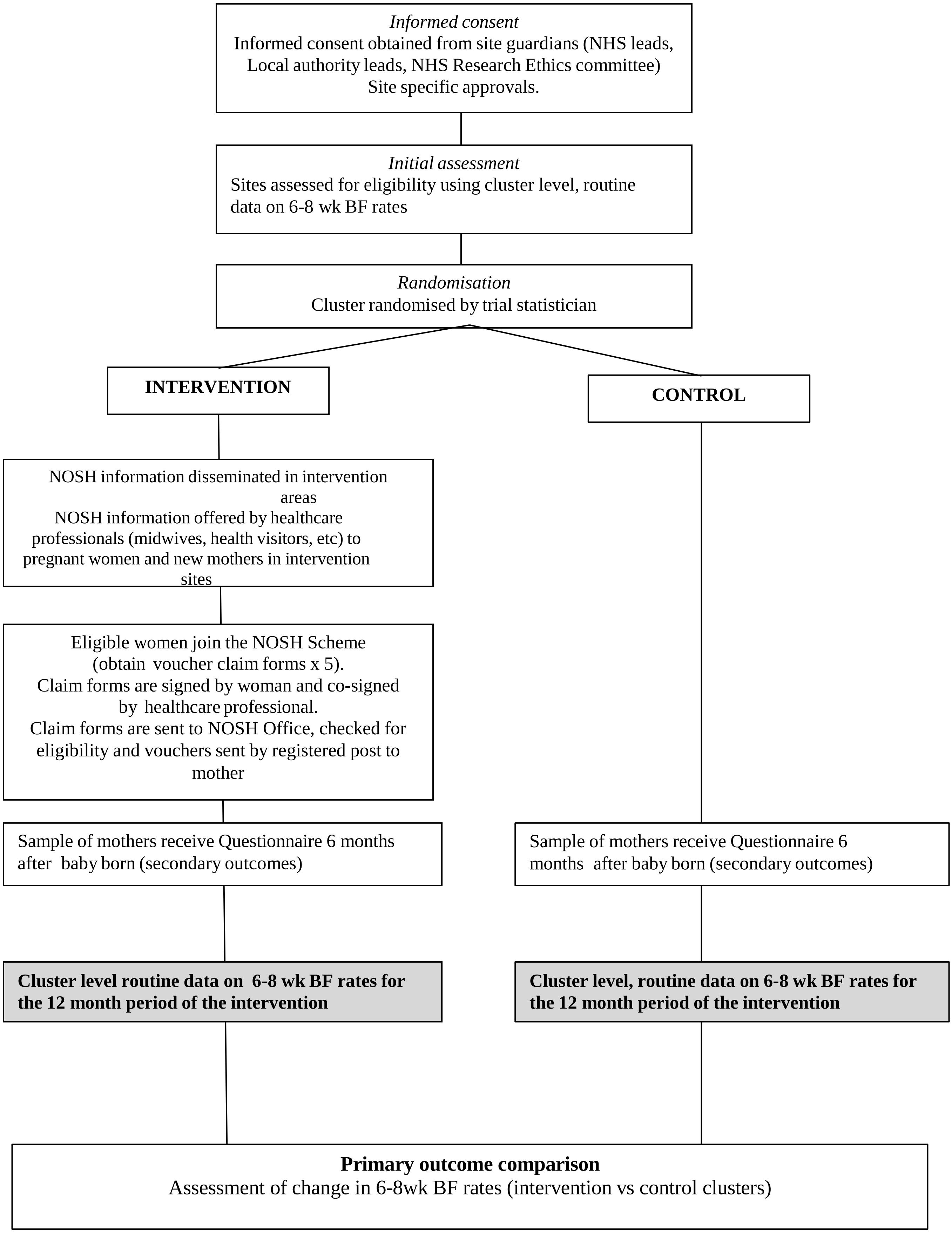

2. Introduction

2.1 Background

2.1.1 Not breastfeeding is a major public health problem

Breastfeeding (BF) is an important factor in the prevention of disease and promotion of health in both infant and mother in the short and long term. Yet BF rates in the UK are among the lowest worldwide, resulting in increased preventable illness for children and mothers and substantial associated costs to the health service. Because infant feeding is socially patterned, low BF rates have a serious negative impact on inequalities in health.

2.1.2 Financial incentives for BF

Financial incentives have been shown to be effective in promoting positive health behaviours. Financial incentives for breastfeeding have been routinely provided in the Quebec region of Canada since the late 1990s. Mothers on benefits receive a monthly 'nursing benefit' of $55 per month until their baby is one year old if they choose to breastfeed their baby (see Appendix 1)[1]. Although BF initiation rates in the Quebec province rose from 45% (1995) to 85% (2006) after the introduction of the 'nursing benefit' (Groleau 2013), there has been no evaluation of the scheme, so this increase cannot be credited to the introduction of the 'nursing benefit'.

If financial incentives are found to be acceptable, effective and cost-effective in promoting BF in the UK, then this will have direct relevance to future UK health policy. Renfrew et al (2012) found that over £17 million could be saved each year in England, through reduced hospital admissions and fewer visits to GPs, from 4 acute conditions (gastrointestinal infection, acute otitis media, lower respiratory tract infection and necrotising enterocolitis) if exclusive breastfeeding rates were to increase to 45% at 4 months and if 75% of babies were fed breast milk at discharge from neonatal units. For each annual cohort of first time mothers, Renfrew et al (2012) also estimated life-time gains from reductions in breast cancer worth over £41million.

2.1.3 The need for a multi-centre trial

BF is a White Paper public health policy priority, and given its potential to impact on health inequalities (e.g. mortality, obesity, early years development), 6-8 week BF rates is an outcome in the Public Health Outcomes Framework (http://www.phoutcomes.info/).

BF is a Department of Health "Vital Signs" target and one of 20 key NHS operating plan performance measures. There are therefore strong drivers within the NHS for the identification of successful strategies to improve BF rates (and the continued collection of BF data).

The NOSH project (Development stage, Feasibility stage and Cluster RCT stage) aims to evaluate the feasibility, acceptability and effectiveness (both clinical/public health and cost effectiveness) of a financial incentive based intervention designed to increase BF at six weeks (and potentially up to six months) in areas with low 6-8 week rates. The results of the project will be used to inform commissioners and other public health decision makers as to the acceptability, feasibility and cost effectiveness of behaviour change support in the form of financial incentives to mothers to breastfeed in areas with low BF rates.

---

1 http://emploiquebec.gouv.qc.ca/publications/pdf/SR_depliant_Prestations_Speciales_en.pdf

If effective, the incentive could have a major impact on the long-term health of the population, reducing the risk of disease in infancy, childhood and adulthood.
If the intervention is shown to be effective at 6-8 weeks, a long term follow up project will be proposed to examine the health and behavioural outcomes for the babies and health outcomes for their mothers. The NOSH project offers a rare and valuable opportunity to examine health outcomes related to infant feeding in the context of a randomised controlled trial (RCT).

2.1.4 Development and Feasibility stages

The three stages of the NOSH project (Development, Feasibility and Cluster RCT) each inform the design of the next. This section briefly describes the findings from the development and feasibility stages.

2.1.5 Development stage findings

Articles reporting the aims, methods and results of the development stage have been published by BMC Pregnancy & Childbirth (Whelan B et al, 2014), and accepted for publication by 'Practising Midwife' Whitford H et al, 2014). Findings from this stage have been presented at UK conferences focusing on behaviour change methods (Relton C et al, 2013; Whelan B et al, 2013). The main outputs of the development stage were: (i) broad acceptance of the idea of financial incentives to BF by both local health providers and local women in areas with low BF rates, (ii) an acceptable intervention that could be tested in the local area, and (iii) agreement and approval by local guardians to test the intervention in the local area in a feasibility study and cluster randomised controlled trial (RCT).

2.1.6 Feasibility stage findings

The Feasibility Stage addressed the questions as to whether or not the intervention (as designed) and the trial methods were acceptable and implementable in the real world. We briefly report the main results and the changes made to the trial design in this section.

The **main findings** from the three feasibility sites in relation to the **intervention** were that:
(i) HCPs alert women about the intervention (NOSH Scheme) and co-sign application forms and voucher claims
(ii) Women apply and send in voucher claims. Over half of all eligible women (54%, n=58/108) applied to join the voucher scheme and over a third (34%, n=37/108) of all eligible mothers claimed 6-8wk vouchers
(iii) In comparison to high street and supermarket vouchers, local shop vouchers were very unpopular with women, therefore these have been withdrawn.

The **main finding** in relation to the **trial methods** was that, despite changes in the responsibility for collecting and reporting routine 6-8wk data on BF (primary outcome measure), this data will be collected, reported and made available for the trial through Public Health and Child Health Information services.

2.1.7 Changes as a result of the feasibility stage

Changes to the intervention

(i) The financial incentive will be provided in the form of supermarket and high street vouchers (not local shop vouchers)

(ii) The wording of 'concerns cards' is the responsibility of local HCPs (as opinion on correct wording may vary between areas). Local HCPs did not want to change the title 'concerns cards'.

Changes to the trial design

(iii) A **4%point increase** in 6-8 week BF rates between the intervention and control clusters has been chosen as the minimally important effect size. This has increased the number of clusters (electoral wards) required for the trial. See section 9.1 for details of the sample size calculation.

(iv) We have widened the sampling frame to include Sheffield, Rotherham, North Derbyshire (which includes Chesterfield), Doncaster, Bassetlaw, providing a total of 82 clusters (wards) with a mean cluster size of 152 (Table 9)

(v) Based on the most recent available data we have estimated an ICC (intra class correlation coefficient) of 0.01.

(vi) The baseline data collection period (see section 8.2.3) has been broadened to take into account differences in the availability in ward-level baseline data between local authority areas. Due to differences between local authorities in the time periods for which baseline data are available, randomisation will be stratified by local authority.

(vii) The cluster-level inclusion criterion with respect to 6-8 week BF rates is now <40%, rather than <=40%

(vi) The timetable has been revised to take into account the delay in the trial start date (Figure 8.2.3).

2.2 The NOSH Trial

The aim of the NOSH Trial is to compare the offer of financial incentives in areas with low BF rates to no offer. The trial design is an open pragmatic cluster randomised controlled trial (RCT) with (a) mixed methods process and context evaluation and (b) a cost effectiveness evaluation.

Clusters are randomised to either Offer of the NOSH financial incentive scheme or No Offer. The intervention is the delivery of the NOSH financial incentive scheme to a cluster (ward). The control clusters receive no delivery of the NOSH financial incentive scheme.

2.2.1 Primary objective

- To test the impact of the intervention (NOSH Scheme) on BF rates at 6-8 weeks in clusters with low BF rates (<40% at 6-8 weeks). The range in BF rates in the eligible wards is 12%-39%. The mean BF rate in these wards is 28%.

2.2.2 Secondary objectives

To test the impact of the intervention on BF rates at initiation (2 days of age), 3 months of age and 6 months of age in electoral wards with low BF rates (<40% at 6-8 weeks)

To examine the cost effectiveness of the intervention (incremental cost per percentage point change in BF) at initiation, 6-8 weeks, 3 months and 6 months in wards with low 6-8 week BF rates (<40% at 6-8 weeks).

To examine the interaction between the effectiveness of the intervention and a range of ward level characteristics, including age, ethnicity and socioeconomic deprivation.

To estimate the incremental cost-effectiveness of the NOSH intervention over an extended time horizon, using an economic model based analysis. This analysis will be based on

health-care costs and health effects that are associated with a range of conditions in the child that are related to infant-feeding patterns.

2.3 Trial activation

The NOSH Trial Steering Committee will ensure that all trial documentation has been reviewed and approved by all relevant bodies and that the following have been obtained prior to activating the NOSH Trial: Research Ethics Committee (REC) approval, adequate funding, confirmation of sponsorship, adequate insurance provision.

3. Selection of clusters (electoral wards)

3.1. Sampling frame & recruitment

The sampling frame for the trial is all electoral wards with low BF rates (< 40% at 6-8 weeks) in the areas of Sheffield, North Derbyshire, Rotherham, Doncaster and Bassetlaw.

The NOSH Scheme is a cluster level intervention designed to increase ward level BF rates. Delivery of the intervention to the clusters and individual women in those clusters is facilitated by healthcare providers (professional and voluntary groups/agencies) involved in the provision of infant feeding support services being informed about the NOSH Scheme and those with key roles (midwives, health visitors and BF peer support workers) being fully briefed.

Recruitment of the NOSH Scheme clusters requires consent by the ‘guardians’ of the study wards - lead representatives of all healthcare providers in the area. This is evidenced by obtaining NHS and local authority Research Governance permission and Research Ethics Committee approvals for the study areas and area healthcare providers to participate.

3.2 Inclusion criteria at the area level

Areas are defined as a local government council (e.g. Metropolitan Borough Council, City Council or a County Council. The following criteria must be met for an area to participate in the NOSH trial:

- have wards with low BF rates (< 40% at 6-8 weeks).
- not be providing financial incentives to breastfeed
- provide all relevant approvals for midwives and health visitors to help deliver the NOSH Scheme (alert and inform women about the NOSH Scheme, co-sign application forms and co-sign claim forms)

3.3Areas and clusters identified

Five local authority areas are included in the trial: Sheffield, North Derbyshire, Rotherham, Doncaster and Bassetlaw. Within these areas, the most recent breast-feeding rate has

been obtained for each ward. Table 3.3a describes the number of clusters (wards) and the number of eligible clusters (wards) in each area.

Table 3.3a Area information

**Areas**	**Eligible wards (<40%) / all wards**
**North Derbyshire**	35* / 77
**Sheffield**	9* / 28
**Rotherham**	20* / 21
**Doncaster**	18 / 21
**Bassetlaw**	11 / 11
**TOTAL**	**82 /147**

- Excluding 3 sites where the intervention was tested during the feasibility stage (Sheffield's Manor Castle ward which includes Manor neighbourhood, Maltby ward in Rotherham and Rother ward in N Derbyshire)

Table 3.3b describes the eligible wards with regards to the mean 6-8wk BF rate for each area (and the range), plus the total number of births per annum in each area and the mean number of births per ward within each area (based on most recent data) .

Table 3.3b BF rates and births for all eligible wards (clusters) by area

**Areas** (number of eligible wards)	**Mean 6-8wk BF rate (range)**	**Total number of births per annum for each area (ward mean)**
**North Derbyshire** (35)	29.9%; (14.8-39.8%)	3273 (n= 93)
**Sheffield** (9)	35.8%; (22.0-39.6%)	2099 (n= 233)
**Rotherham** (20)	22.7%; (18.2-27.3%)	3019 (n= 150)
**Doncaster** (18)	26.0%; (11.8-39.1%)	3315 (n= 184)
**Bassetlaw** (11)	31.0% (27.0-36.5%)	792 (n= 72)
**TOTAL** (82)		**12498 (n=152)**

3.4 Area initiation & activation

When the inclusion criteria (section 3.2) are met then the NOSH Research team will implement induction visits to all midwife and health visitor teams in the trial areas. After randomisation (when the individual control and intervention clusters have been identified), the NOSH Research Team will distribute all relevant information (postcodes of intervention clusters and NOSH booklets) to the relevant midwifery and health visitor teams.

4. Informed consent

4.1. Obtaining informed consent from areas

Area consent is obtained from the lead organisations of those healthcare professionals involved in delivering infant feeding services. Consent (i.e. approvals) for all eligible clusters to participate in the cluster has been obtained by cluster guardians – those who help deliver infant feeding services in each area (local NHS and Local Authorities organisations). Consent was obtained for the implementation of the feasibility study and the cluster RCT at the same time (NOSH Protocol V1.0). Table 4 lists the consents/ approvals obtained or pending.

Table 4 NOSH Research Consents/Approvals Status Summary 10.10.14

Organisation/Authority	Final Decision	Date of decision
NRES	Approved	26/07/2013
CLRN/Portfolio Adoption	Approved	16/09/2013
NHS R&D (STH)	Accepted	
**Site specific forms**		
Sheffield Teaching Hospitals (midwives)	Approved	18/10/2013
Sheffield Children's Hospital (health visitors)	Approved	17/09/2013
Rotherham Foundation Trust (midwives & health visitors)	Approved	12/12/2013
Chesterfield Royal Hospital (midwives)	Approved	07/11/2013
Derbyshire Community Health Services (health visitors & BF peer support)	Approved	30/10/2013
Doncaster & Bassetlaw Hospitals (midwives)	Site specific forms in process.	
Rotherham, Doncaster and South Humber NHS Trust	Site specific forms in process.	
**Local Authorities (AREAS)**		
Sheffield CC	Approved (Accept PHE funding for vouchers only)	05/09/2013
Derbyshire CC	Approved	21/10/2013
Rotherham MBC	Approved	30/08/2013
Doncaster MBC	Approved	9/10/14
Bassetlaw (part of Nottingham CC)	In process	

4.2. Obtaining informed consent from individuals

4.2.1 Consent to participate in the NOSH Scheme

Participation in the NOSH Scheme is voluntary. Women are able to freely join and leave the NOSH Scheme.

4.2.2 Consent for data collection at the individual level

A purposive sample of **Healthcare providers** (professional and voluntary groups/agencies involved in the provision of infant feeding support services) from the wards will be identified by the research team in liaison with local collaborators from participating NHS Trusts and Local Authorities. A critical case sampling approach will be used to select participants based on criteria that are seen to be important in understanding how the scheme worked in practice and outcome data such as changes in breastfeeding rates. Staff will be approached by the research team and invited to participate in either a face to face focus group or interview about their views and experiences of the NOSH Scheme. The researchers will provide an Information Sheet about the study and participants will be asked to sign a consent form prior to being interviewed.

For research about the NOSH Scheme, **women (and sometimes their partners)** will be identified and approached twice by their health visitors:

(i) A purposive sample of women eligible for the NOSH Scheme (and their partners) will be approached by health visitors and contact details of those who consent will be passed to the researchers who will then send an Information Sheet and invitation to participate in an interview or focus group about their views and experiences of the NOSH Scheme. All participants will be asked to sign a consent form prior to being interviewed. Participants remain free to refuse or withdraw at any time from the study without giving reasons.

(ii) In both control and intervention areas a sample of women who have 6 month old babies will be approached by their health visitor and asked to complete a **Mother and Baby 6 month Questionnaire.** This short simple questionnaire will be provided both in paper and online format and will be provided with an information sheet about the research (on the front page of the questionnaire). The return of questionnaires to the researchers will be taken as implicit consent for the data to be used for the purposes of the study. Only if participants are willing to be contacted again by the research team will their contact details and a signature of their willingness to be contacted again sought.

5. Selection of individual eligible women

5.1. Trial inclusion and exclusion criteria

Inclusion criteria: All women aged at least 16 years old, ordinarily resident in each ward cluster and with a pregnancy due date before the end of the NOSH Claim period will be eligible to apply to the NOSH Scheme.

Exclusion criteria: No exclusion criteria are stipulated. However, healthcare professionals have the right to not offer the NOSH Scheme to women as they see fit (e.g. women transferring in and out of wards, medical conditions affecting BF, alcohol/drug abuse, babies in special care etc.). The extensive consultation with healthcare providers did not produce any definite exclusion criteria, instead healthcare providers wanted to make decisions on a case by case basis.

5.2. Data collection at the individual level

For data collection at the individual level about the NOSH Scheme the same inclusion criteria apply for HCPs and women as for NOSH Scheme (see above). Health visitors will ensure that no women will be contacted to participate in research about the NOSH Scheme if their baby has died or is seriously ill.

6. Randomisation procedure

Randomisation and allocation of control and experimental clusters (electoral wards) for the cluster RCT will be stratified by local authority area. Randomisation and allocation will be undertaken by an independent statistician at the University of Sheffield.

7. Trial intervention

7.1 Summary

The intervention to be trialled is the NOSH Scheme (Appendix 3). This is an area level behaviour change intervention in the form of the offer of a structured financial incentive (shopping vouchers each worth £40 x 5) to mothers. The intervention is designed to increase BF rates at 6-8wks in wards with low BF rates.

7.2 Delivery of the intervention

This section describes how the intervention is introduced into the cluster communities, the induction of local healthcare providers who will help deliver the intervention, how women hear about the NOSH Scheme and how they can apply to join, how women claim the vouchers and the types of vouchers they can receive. The intervention flowchart describing how the intervention is delivered is shown in Figure 7.1

Figure 7.1 Delivery of the NOSH Scheme

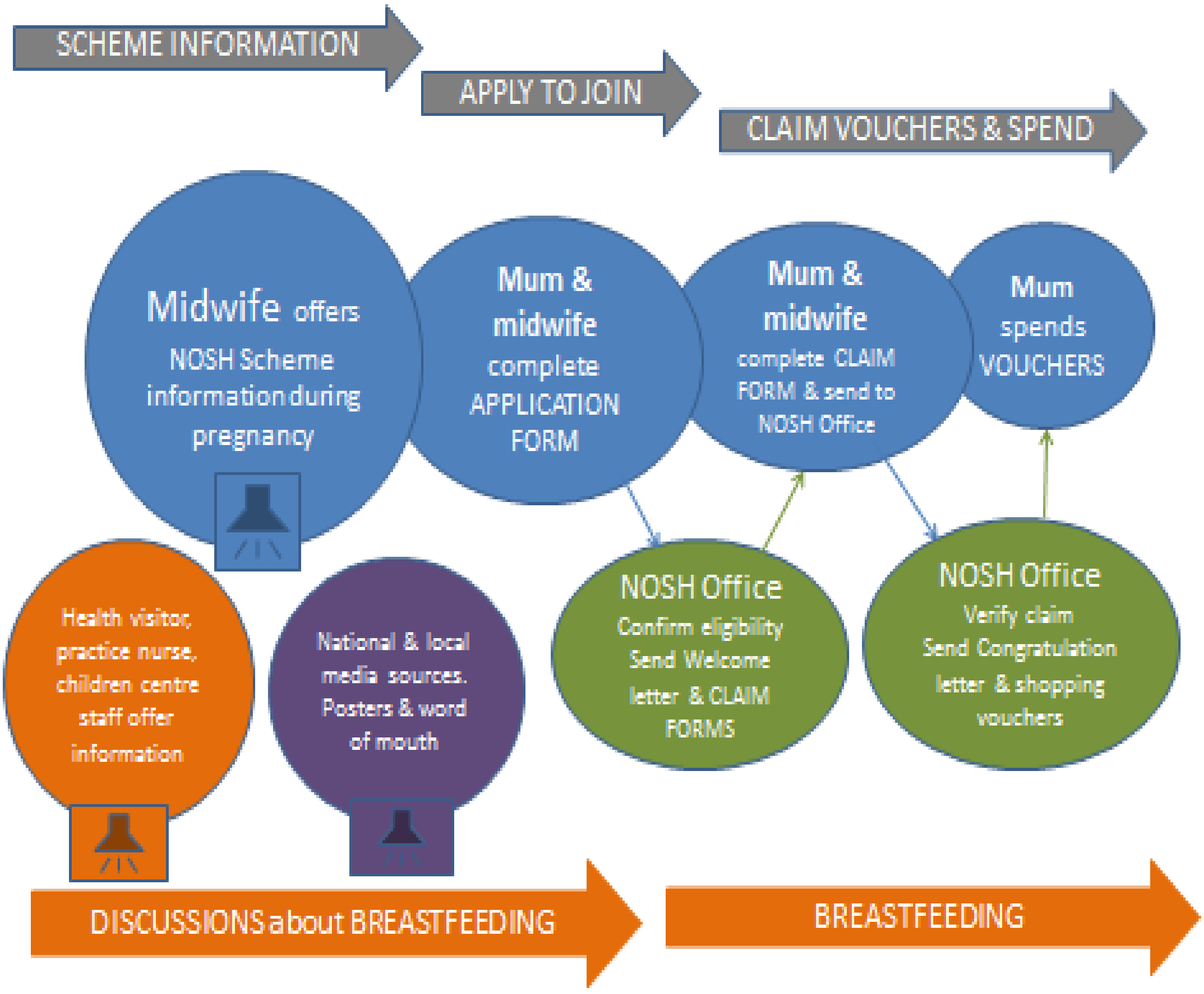

7.2.1 Introduction of the NOSH Scheme into the cluster communities

The intervention will be introduced into the clusters randomised to the Offer Group in multiple ways:

- Information about the NOSH Scheme (posters, booklets etc.) distributed in Children's Centres, GP surgeries, post offices and other public places in the cluster area alongside a press release to the local and national media.
- Briefing notes and invitations to attend induction sessions sent to all Health Care Providers (HCPs) in the intervention clusters (co-led by local HCPs and the NOSH researchers in locations convenient to local HCPs)

7.2.2 Induction of healthcare providers

The induction sessions will familiarise HCPs with the NOSH Scheme

NOSH Scheme Guidelines for Healthcare providers

The NOSH Scheme is designed to complement existing support and guidance on infant feeding provided by midwives, health visitors, BF support workers and other healthcare providers

Alert and inform individual eligible women about the NOSH Scheme during their routine HCP visits/ clinics including giving NOSH Booklets
Co-sign the NOSH Scheme Application form (in NOSH Scheme Booklet) and NOSH Scheme Claim Forms on request from eligible women
Note concerns about mothers reported BF using the NOSH Concerns Card. Any concerns reported will not affect the woman's receipt of vouchers or her care. Analysis of the Concern Cards will inform understanding of the acceptability and reliability of the verification method.

7.2.3 How women join the NOSH Scheme

To join the NOSH Scheme women will fill in Part A of the NOSH Application form in the NOSH Booklet (Appendix 3). (Part B will be co-signed by her midwife or other HCP if needed). The completed Application Form will then be sent to the NOSH Office (at the University of Sheffield). The NOSH Office will send a 'Welcome Pack' (NOSH Scheme Welcome Letter, five NOSH Claim Forms for vouchers each worth £40, NOSH fridge magnet, NOSH pen, NOSH postcards and NOSH stickers with contact details of local BF advice and support). Women will be encouraged to put their NOSH Claim Forms and NOSH stickers in the NOSH fridge magnet. After the birth we recommend mothers put the Claim Forms inside their Child Health Record (Red Book).

Figure 7.1 How women hear about the NOSH Scheme and join

NOSH induction sessions with HCPs and Children Centre managers
NOSH Scheme posters
NOSH Scheme Booklets distributed to HCPs (midwives, health visitors, BF support workers, GPs and practice nurses)

Midwife informs woman about the NOSH scheme during a routine antenatal visit
BFPSW reinforces information from Midwife during routine antenatal visit(s)
Midwife AND BFPSW offer woman the NOSH Booklet
Woman completes Application Form Part A
Midwife completes Application Form Part B
Woman posts Application Form to NOSH Office

NOSH Office confirms eligibility
Women not eligible sent letter/ phoned from NOSH Office
NOSH 'Welcome Pack' sent including

- Welcome letter
- 5 NOSH Claim Forms (with SAE) for Shopping Vouchers
- NOSH clip fridge magnet with BF support contact details

7.2.4 How women claim NOSH Vouchers.

Women sign and date each NOSH Claim Form stating that her baby is currently receiving breast milk. Claim Forms can be signed when the baby is 2+ days, 10+ days, 6+ weeks, 3+ months and 6-7 months. Mothers will ask their local healthcare provider (HCP) to co-sign the NOSH Claim Form. s will then send the co-signed NOSH Claim Form to the NOSH Office and the vouchers will be sent to women by return post.

Figure 7.2 How women claim NOSH Vouchers

Day 2+
NOSH Claim Form signed by (i) mother AND (ii) Midwife or Health Visitor Mother posts signed Claim Form to NOSH Office
NOSH Office verifies Claim Form & sends vouchers to mother (Total value £40)

Day 10+, Week 6, Week 13 and Week 26
NOSH Claim Forms signed by (i) mother AND (ii) Midwife or Health Visitor or BF support worker
Mother posts signed Claim Form to NOSH Office
NOSH Office verifies Claim Form & sends vouchers to mother (Total value of each voucher £40)

7.2.5 Type of vouchers

Women can claim local supermarket vouchers or Love2shop (redeemable in chain high street shops) vouchers or a combination of both (£20 of each).

7.3 Verification of breastfeeding (BF)

This section describes a number of different approaches to verification of breastfeeding including the approach used in Quebec, the UK routine data collection perspective, local stakeholder views on verifying BF. The question as to how one might verify that a mother is breastfeeding has been considered at length by the research team and those who we consulted during the development and feasibility stages. The locally acceptable method is the method which will be used in the trial.

7.3.1 Verification of BF in Quebec 'Nursing Benefit' Scheme

As mentioned in section 2.1.2, financial incentives for breastfeeding have been routinely provided in the Quebec region of Canada since the late 1990s. If women choose to breastfeed, mothers on benefits receive a monthly 'nursing benefit' of $55 each month until their baby is one year old (Appendix 1). The verification method used in the Quebec scheme relies on women's self-report. To join the scheme, women ask their doctors to sign a statement that they are pregnant and intend to breastfeed. Mother
s are asked to notify the local employment agency if they stop breastfeeding. No other method of verification is used.

7.3.2 UK routine data collection and verification of BF for outcome measurement

We will use routine data to determine cluster-level 6-8 week BF prevalence. These data are based on healthcare professional judgement, after discussion with the mother. The routinely reported 6-8 BF rates are essentially based on mother's self-report.

7.3.3 Local stakeholder views on verifying BF

The consensus from the extensive consultation with local stakeholders during Development Stage of the NOSH Project was that verification of BF should be confirmed by both a signed statement from the mother and a signed statement from their healthcare provider on the Voucher Claim form.

7.3.4 Method of verifying BF in the trial

Verification of BF will be by mothers self-report via a signed statement, with co-signing by healthcare professional. This is in line with the method for determining the trial primary outcome, and in line with stakeholder views.
If a mother is BF, she will sign the following statement on the NOSH Claim Form:

"I confirm that my baby is receiving breast milk".

The healthcare professional will be asked to co-sign the form with these statements:

(1) I am advised by …………………that she is breast feeding
and I am signing this form on that basis, and
(2) I have discussed BF with mum today.

There is a separate claim forms for the time points when the baby is 2 days old, 10 days old, 6 weeks old, 3 months old and 6 months old.

If a healthcare professional has concerns that the baby is not receiving breast milk, then a separate "expression of concern form" can be submitted direct to the NOSH office with the details of the concern. Submission of an expression of concern form does not mean that the healthcare provider either should or should not sign the NOSH Claim Form

Healthcare providers are involved in both the delivery of the intervention and the verification of whether or not women are breast feeding therefore, it is vital that the NOSH Scheme does not compromise the relationship between the mother and the healthcare provider. Therefore, if the healthcare providers has concerns that a woman is not BF (but claiming that she is), then healthcare providers will sign the Claim Form and then will send a record of any concerns they experience to the NOSH team. All claims will be paid (regardless of whether concerns have been expressed). Analysis of the Expression of Concern forms will inform understanding of the reliability of the verification method.

7.4 Impact on benefits

We are advised by CPAG (Child Poverty Action Group) that NOSH vouchers will be treated as charitable or voluntary payments thus they will be disregarded for means-tested benefits; as non-taxable income they will therefore be disregarded for tax credits; and capital which will not affect entitlement to benefits or tax credits.

7.5 Duration of the intervention

Women who have babies born during the 12 months (planned to be 17.2.2015-17.2.2016 will be eligible to join the scheme but the NOSH Scheme will be publicised in the intervention cluster from two to three months beforehand.

7.6 Role of the NOSH Office

The administration of the NOSH Scheme for the trial will run by the NOSH Office within the NOSH research team in ScHARR at the University of Sheffield. The NOSH Office will:

- Develop and maintain a secure administrative database to manage all applications and claims and to provide basic monitoring and reports from this data, in line with data protection requirements
- Provide secure storage, processing and handling of all vouchers, in line with UoS Finance Department recommendations
- To order and maintain an adequate stock of appropriate vouchers to ensure timely turnaround of claims, liaising with University procurement and suppliers as required
- Provide a point of contact for the public and professionals enquiring about the scheme via telephone and email. Triaging enquiries to ensure appropriate and timely responses
- Ensure secure storage of all project paperwork
- Monitor voucher expenditure against budget
- Administer day-to-day management of the Voucher Scheme (assess Application forms for eligibility to the NOSH Scheme (check HCP names and signatures, postcodes); send out NOSH Welcome Packs (including NOSH Claim Forms), check and process NOSH Claim Forms and send out NOSH Vouchers; collate NOSH Concerns Cards; arrange traceable postage via Royal Mail so that delivery can be tracked, monitored and any issues addressed.

8. Data collection

8.1 Units of data collection, and definition of cluster areas

We define the following units of data collection: mother, baby and cluster area. The trial is cluster randomised, with randomisation at cluster level.

8.2 Primary outcome measure

The primary outcome is the change in cluster-level 6-8 week BF prevalence between baseline and the end of the trial.

8.2.1 Definition of BF
Six to eight week BF is defined as the BF of a baby at 6-8 weeks of age, as assessed by a healthcare professional for the purposes of the 6-8 week BF routine data collection exercise.

8.2.2 Unit of data collection and definition of clusters
Data for the primary outcome measure will be collected at cluster (electoral ward) level. All clusters can be identified by postcode.

8.2.3 Definition of periods of baseline (pre-trial) and during-trial data collection & timetable

The intervention is planned to be offered to mothers with babies born between 17.2.15 and 17.2.16. A timetable showing the eligibility and delivery periods of the intervention is shown in Figure 8.2. .

The **baseline data collection period** for the primary outcome (ward-level 6-8wk BF rate) has been defined as prior to **01.04.13**

In setting the baseline data collection period there were three considerations. Firstly, it was not possible to set the baseline data collection period any later than 01.04.13 as the datasets submitted to the DH were not complete in the period following this date due to national changes in the way information was collected following the implementation of the Health and Social Care Act. Secondly, the baseline data collection period needed to be **before** January 2013 in order to avoid any contamination effect of the pre-trial development and feasibility stages of the study. Thirdly, data completeness in the time period between 2008 and 2013 varied between local authorities (see Table 8.2.3). For example, for Rotherham, BF rates are not available at electoral ward level after 2010 (though they are available at GP level).

To allow for any effect of this variability, randomisation is stratified by area, and the period of baseline data collection will be added as a covariate in analyses that include baseline adjustment (see sections 8.3 and 9.2).

Table 8.2.3 Baseline data collection periods for each area

AREA	Baseline data collection period	Type of area
**Sheffield**	2010/11-2011/2012	City Council
**North Derbyshire**	2012/13	County Council
**Rotherham**	2008-2010	Metropolitan Borough Council
**Bassetlaw**	2012/13	County council
**Doncaster**	2012/13	City Council

The “baseline rate” is thus defined as the cluster-level period prevalence of 6-8 week BF, in any of the 12 or 24 or 36 month period prior between 01.01.08 to 01.04.13. The “during-trial rate” is defined as the cluster-level period prevalence of 6-8 week BF, in the 12 month period during which the financial incentive scheme is offered in the intervention clusters.

Figure 8.2 Timetable for trial and intervention delivery

	Nov **2014**	Dec 2014	Jan **2015**	Feb 2015	Mar 2015	April 2015	May 2015	June 2015	July 2015	Aug 2015	Sept 2015	Oct 2015	Nov 2015	Dec 2015	Jan **2016**	Feb2016	Mar 2016	Apr 2016	May 2016	Jun 2016	Jul 2016	Aug 2016
**Trial schedule**																						
TRIAL ELIGIBILITY (based EDD, DOB)				■	■	■	■	■	■	■	■	■	■	■	■							
‘DURING-TRIAL’ routine BF data collection*						■	■	■	■	■	■	■	■	■	■	■	■					
**Intervention delivery schedule**																						
HCP induction sessions	■	■	■																			
Dissemination of NOSH info	■	■	■	■	■	■	■	■	■	■	■	■	■	■	■							
Scheme application window	■	■	■	■	■	■	■	■	■	■	■	■	■	■	■	■	■					
2 day voucher claim period				■	■	■	■	■	■	■	■	■	■	■	■							
6-8wk voucher claims period						■	■	■	■	■	■	■	■	■	■	■	■					
6 month voucher claims period										■	■	■	■	■	■	■	■	■	■	■	■	

***** *To be used to calculate primary outcome (cluster 6-8wk BF rates)*

8.3 Covariate data collection

Table 8 lists the quantitative data on covariates that we will collect.

Table 8.3 Mother, baby and cluster level covariates

Data item	Unit of data collection	Data source	Data collection time point
Cluster level covariates			
Cluster level deprivation (IMD 2010)	Cluster	Department for Communities + LA*, via local data sources	2010
Cluster level age and ethnicity	Cluster	Census, via local routine data sources	2013-2014
Birth rate	Cluster	Census, via local routine data sources	2013-2014
Maternal smoking rate at delivery	Cluster	Local routine data sources	2013-2014
Time period during which baseline 6-8wk BF rate data was collected	Area	PH and Child Health Information Services	2008-2013
Individual level covariates			
Mother's age	Mother	Mother self-report questionnaire	6 months after baby's birth
Mother's ethnicity	Mother	Mother self-report questionnaire	6 months after baby's birth
Mother's smoking status	Mother	Mother self-report questionnaire	6 months after baby's birth
Is the mother claiming any benefit	Mother	Mother self-report questionnaire	6 months after baby's birth
Number and age of Mother's other children	Mother	Mother self-report questionnaire	6 months after baby's birth
Birth weight	Baby	Mother self-report questionnaire	6 months after baby's birth

*https://www.gov.uk/government/publications/english-indices-of-deprivation-2010

8.4 Secondary outcome measures

Secondary outcomes are listed in table 8.4

Table 8.4 Secondary outcome measures

Secondary outcome	Unit of data collection	Data source	Data collection time point
Cluster level outcomes			
BF initiation	Cluster	Routine data	"Baseline" and "during-trial" (as defined above)
Individual level outcomes			
Duration of exclusive BF	Baby	Mother self-report questionnaire	6 months after baby's birth
Duration of any BF	Baby	Mother self-report questionnaire	6 months after baby's birth
Number of consultations with health	Baby	Mother self-report	6 months after baby's

professionals concerning: gastrointestinal tract infection, otitis media, respiratory tract infection, atopic eczema		questionnaire	birth
Number and length of admissions to hospital with: gastrointestinal tract infection, otitis media, respiratory tract infection, atopic eczema	Baby	Mother self-report questionnaire	6 months after baby's birth

8.5 Sampling strategy for obtaining mother's self-report data at six months

The "six-month" questionnaire will be sent to two independent cohorts of eligible mothers in intervention and control clusters during two, two-week time periods. The first two-week time period will be in April 2015 and the second will be in October 2015. Eligible mothers are defined as those mothers whose baby was born in a trial cluster, and whose baby reaches the age of six months sometime in the two-week period. Using this sampling strategy we expect the number of eligible mothers to be in the region of 450-500. The number of babies will be slightly larger than the number of mothers due to the presence of multiple births. In cases of multiple birth mothers will be asked to complete questionnaires for each baby.
This number should be sufficient to provide the information required (and be more time and resource efficient than targeting the whole trial population).

Letter inviting mothers to complete the short questionnaire will be sent to them by their health visitor. The letter will invite them to either complete a paper questionnaire that is included with the letter, or alternatively telephone the NOSH Office to answer the questions over the phone with a member of the research team. All mothers who complete the questionnaire will be sent a £5 love2shop voucher. The cost of vouchers for the "six month" questionnaire is expected to be less than £2,500. All mothers who complete the questionnaire will be asked if they consent to further contact in relation to the NOSH project.

8.6 Risk to accessing primary outcome data

In order to mitigate any risk to 6-8 week data collection at this time we are building and retaining good links with local government Public Health.
We are aware that with the transfer of the commissioning of health visiting from NHS England to public health in local authorities in 2015 (October 2015), this may impact on the trials ability access to routine 6-8 week BF data collection. However, we have good reason to have confidence that national systems are being set up to ensure good data collection for 6-8 week BF prevalence. These should steadily improve as contract management by NHS England of HV providers to the national specification becomes more robust. The Health Visitor national spec includes outcomes around 6-8 week BF prevalence and data coverage (See Appendix 2). As BF is an indicator in the Public Health Outcomes Framework, and PH in local government will remain accountable for that, it is extremely unlikely they will stop commissioning HVs to collect that data (see page 51 of PHOF for the BF indicator[2]).

[2]https://www.gov.uk/government/uploads/system/uploads/attachment_data/file/263662/2901502_PHOF_Improving_Outcomes_PT2_v1_1.pdf

Moreover, four of the five areas have achieved BFI Stage 3 status. In order for them to retain this status 6-8wk BF data must be collected. Rotherham does not have BFI status, however, there is currently a CQUIN (an incentive payment for HVs) to ensure that 80% of all mothers BF at 14 days continue to BF to 6-8wk – and this requires 6-8wk BF data collection.

8.7 Intervention process monitoring

The monitoring of the process and delivery of the intervention will be conducted using both qualitative and quantitative methods.

8.7.1 Qualitative data collection

Individual level data will be sought on the views of HCPs, commissioners, funders and policy makers regarding the process of delivering the intervention and the completeness and accuracy of the routinely collected 6-8wk BF using interviews and focus groups with the researchers. Both HCPs involved and not involved in the implementation of the NOSH scheme will be sought (particularly their views on the impact of the scheme on their work and the reporting of the routinely collected 6-8 week BF data). Topic guide refinement, sampling strategies and data collection and analysis will be iterative to address the specific research questions.
Awareness of the intervention and views and experiences of the intervention will be collected using interviews and focus groups with mothers (and their partners) when their babies are 10+ weeks old.

8.7.2 Quantitative data collection

For those who take up the NOSH Scheme, information on all applications and claims will be collected and monitored by the NOSH Office.
Cluster level descriptive data will be collected using routine demographic and deprivation data for each cluster.
Information about dissemination and awareness of the NOSH Scheme will be collected via the 6 month mother and baby survey in two samples of mothers in the intervention and control groups.

8.8 Methods to protect against sources of bias

It is likely that knowledge of the intervention (NOSH Scheme) will spread to control clusters during the feasibility and cluster RCT stages. The extent and direction of any ‘contamination’ bias introduced by knowledge of the intervention in the No Offer clusters will be assessed using retrospective quarterly 6-8 week BF prevalence data routinely collected since April 2007. Two samples of women in non-intervention clusters will be asked if they have heard about the financial incentives to breastfeed scheme in the 6 month questionnaires (section 8.5).

9. Statistics & analysis

9.1 Sample size for the NOSH Trial

The ICC (0.01) was estimated from the most recently available 6-8 week BF rates in the clusters in the sampling frame. Based on data from 82 clusters and 12,498 births the mean cluster size was 152 (see Table 9.1). The proportion breast feeding at 6-8 weeks was 27.9% (3496/12498).

Table 9.1 BF rates and births for all eligible wards (clusters) by area

**Areas** (number of eligible wards)	**Mean 6-8wk BF rate (range)**	**Total number of births per annum for each area (ward mean)**
**North Derbyshire** (35)	29.9%; (14.8-39.8%)	3273 (n= 93)
**Sheffield** (9)	35.8%; (22.0-39.6%)	2099 (n= 233)
**Rotherham** (20)	22.7%; (18.2-27.3%)	3019 (n= 150)
**Doncaster** (18)	26.0%; (11.8-39.1%)	3315 (n= 184)
**Bassetlaw** (11)	31.0% (27.0-36.5%)	792 (n= 72)
**TOTAL** (82)		**12498 (n=152)**

In order to reduce the risk of the trial producing a non-significant result, a 4%point increase in 6-8 week BF rates between the intervention and control clusters has been chosen as the effect size.

We have set the minimum important difference as a 4%point increase in 6-8wk breastfeeding rates between the intervention and comparator groups. Assuming an ICC of 0.01; average cluster size of 152 births and a mean 6-8 week breast feeding rate of 26.9% in the control arm (28.9% in the intervention arm): then with 5162 births per group (10324 in total) the trial is powered to detect a 4% point increase in breastfeeding rates (from 28% to 32%) as statistically significant at the 5% (two-sided) level and 80% power. For a cluster RCT this will require 68 clusters to be randomised (152 per group).

Wards will be randomly allocated to intervention or control using 1:1 randomisation strategy with stratification at area level. Eligible wards will be allocated a number with each number corresponding to either ‘control’ or ‘intervention’.

9.2 Public Health effectiveness

We will compare primary and secondary outcomes between the control and intervention group wards.

The primary outcome measure is area-level 6-8 week breastfeeding period prevalence over the time period 1st September 2014 to 31st August 2015. We will access the data that is

collected for the purposes of the Public Health Outcomes Framework, and aggregate this at ward level. We will test for a difference between groups using a chi-square analysis, and we will present adjusted and unadjusted odds ratios from a logistic regression. In the adjusted analysis we will include the following covariates: 6-8 week breastfeeding period prevalence between 1st Sept 2012 and 31st August 2013; maternal age (proportion of mothers under 20); area-level deprivation (IMD 2010); proportion non-White; birth rate; maternal smoking rate).

For the secondary outcomes that are reported in the six month questionnaire we will compare intervention and control groups using random effects models with individuals nested within areas. We will compare the following measures between intervention and control groups: rate of breastfeeding initiation and at 6-8 weeks, 3 months and 6 months; duration of exclusive breastfeeding; duration of any breastfeeding; incidence of GI tract infection, otitis media, respiratory tract infection and atopic eczema. In each case we will present adjusted and unadjusted effect sizes. For the models with adjustment we will include the following individual level covariates: maternal age, maternal ethnicity (White vs Mixed vs Asian vs Black as per ONS definitions); maternal smoking status; maternal parity; maternal benefit claimant status (yes / no); and baby's birth weight. We will include the following area-level covariates: IMD 2010 and birth rate. . We will perform an exploratory subgroup analysis with respect to the primary outcome by testing for an interaction between the intervention group and sub-groups as defined by age, ethnicity and ward level of socioeconomic deprivation.

All analyses will be based on “intention to treat” at cluster level. Statistical tests will be two-sided with significance declared at 5%. All reporting will meet CONSORT statement standards.

9.3 Economic evaluation

The base case economic evaluation will take an NHS perspective. The economic analysis will be two-fold, including; a within-trial cost-effectiveness analysis and beyond-trial modelling of longer term expectations for cost-effectiveness.

The costs to be accounted for include provision of NOSH service and any cost impacts arising from changes in breastfeeding e.g. on health service use and on participants. The trial will collect data on these up to 6 months post birth. As there is no change in service provision in the control clusters we assume costs of the intervention are the true incremental costs of provision. However, the cost impacts on health service provision and participant costs will be compared between intervention and control.

Data collection for the intervention will include: a) costs that don’t vary by cluster or participant (e.g. time spent setting up the negotiating coverage of voucher) that need to be apportioned to clusters b) costs that may vary by cluster but not by individual participant (e.g. induction and training of staff): c) costs that vary by participant (e.g. Number of vouchers sent, contacts made to NOSH office) that can be grouped by cluster. Methods of measuring resource use will be based on; diary reviews week time-sheets and interviews with NOSH team, review of NOSH Office records.

Resource use impacts measured will include health service use (GP visits and hospital admissions) by babies for a range of conditions (GI infections, otitis media, respiratory tract

infections, atopic eczema) and for all other reasons (the latter to allow for participants not being sure of cause). There is good quality evidence (Renfrew et al 2013) that these are influenced positively by breastfeeding. Data will be collected in the 6 month mother and baby questionnaire.

Unit costs will be based on NHS reference costs (DH 2012) and other national averages to increase generalizability (e.g. PSSRU 2013). A breakdown of total costs will be presented by area, alternative viewpoints (i.e. commissioner and participant), and main activity (e.g. set up, training and implementation).

The main outcome of the economic analysis will be cost per percentage point increase in BF rates at area level. The within-trial analysis will comprise a regression analysis to explore variation in costs and effects according to characteristics of areas and participants socio demographic details (Willan and Briggs, 2006). Analyses will account for clustering, correlation between costs and effects and missing data as appropriate (Gomes et al 2012abc, Gomes 2103). Sensitivity and scenario analysis will explore variations in discount rates, perspectives and potential roll-out of the NOSH scheme.

Modelling cost-effectiveness is important where benefits and costs from an intervention extend beyond a trial period – from limitations connected to the time horizon and/or capture of costs and effects. It is of particular relevance to this trial, where the primary outcome is a behaviour change (change in breastfeeding rates) as, to simplify the trial, known costs and effects on disease prevention are either not captured (e.g. necrotising enterocolitis in pre-term babies and breast cancer) or are based on self-reported data from a sample of trial participants (GI tract infection, respiratory tract infection, otitis media). It is also the case that the trial runs for only 6 months and there is good quality evidence of impacts for at least one year (Renfrew et al 2012). While some relevant models exist (e.g. Jacklin et al 2007, Suwantika and Postma 2013, there is limited evidence on cost-effectiveness of interventions to promote breastfeeding and hence few models (Carr et al 2001). We will therefore draw on best available data and modelling to date using evidence with which we are very familiar and adapt the model presented by Renfrew et al (2012) in 3 ways; transfer the models of childhood conditions from static to dynamic models; move from disease cases averted to QALYs (but still provide the breakdown for disease cases averted); and, following an initial modelling using the one year time horizon, explore the possibility of adopting a longer time horizon (e.g. 5 years) subject to data availability.

If results show a trade-off, with NOSH having better outcomes but higher costs, an incremental analysis will be conducted based on cost/QALY and cost/life year. Otherwise, results will be summarised using a net monetary benefit statistic, using NICE thresholds of £20,000 and £30,000/QALY. The effect of uncertainty on model results will be estimated using deterministic and probabilistic sensitivity analysis (PSA) and account for good practices in developing economic models (Caro et al 2012) and guidance on evaluating public health interventions in the UK (NICE 2013). The deterministic sensitivity analysis will examine, for example, the impact of different evidence based assumptions on maintenance of breastfeeding over time. The PSA will jointly estimate the effect of uncertainty emanating from all data sources and assumptions and will be presented using a scatterplot of points on the cost-effectiveness plane – illustrating the possible ranges of estimates of incremental costs and incremental QALYs. Cost-effectiveness acceptability curves will be presented

9.4 Mixed methods process/ context evaluation

Process evaluation is commonly undertaken alongside trials of complex interventions in order to inform the interpretation of the trial results by offering accounts of the delivery of the intervention (fidelity, compliance, and variability in multi-centre trials). This allows the triallists to distinguish between results that are due to the intervention succeeding or failing, and those due to the impact of the implementation of the intervention and is especially valuable in community-based trials (Stapleton et al 2002, Oakley et al 2006). Where a cluster design is used, an additional value resides in the ability of an evaluation to assess the impact of the local context on the implementation of the intervention in each setting (cluster). This is likely to be particularly relevant to trials of public health initiatives (Hawe et al 2004; Hoddinott et al 2010). Process/context evaluations can also be used to explore unintended consequences of the intervention.
Undertaking a process/context evaluation usually entails the collection of qualitative data through key informant interviews (healthcare providers, women and their partners and local policy makers) and observations of the intervention delivery, as well as the collection of quantitative data through the mapping of context level factors (e.g. local resources and demographics such as the characteristics of Children's centres and BFPSWs) (Hawe et al 2004). Both types of data may be used to identify anticipated and emergent factors that impact on the delivery of the trial intervention at the cluster level. We propose to conduct a process/context evaluation alongside the cluster trial in anticipation of variability in the 6-8 week BF rates within the experimental and control cluster groups. We will treat each ward (cluster) as a 'case study' and interview pregnant and postpartum women within each cluster, healthcare providers and funders and commissioners at various stages of the study to capture local characteristics and the impact of any locally-based changes that occur during the research project. Important themes regarding likely locally influenced barriers and facilitators to the implementation of the intervention will be monitored during both the feasibility and cluster RCT stages of the project. Relevant quantitative factors are likely to include the location of a Children's Centre in the wards and local demographics, such as the average maternal age, deprivation status, and ethnic composition of each ward. The two types of data will be combined in analysis to build a model of the contextual factors influencing the implementation of financial incentives for BF. Model-building will be undertaken prospectively, prior to the trial results, and the findings applied retrospectively to explore the possibility that the model can provide an explanation of any observed differences in the trial outcomes between cluster sites and explore underlying causal mechanisms.

9.5 Qualitative data analysis

NVivo software package will be used to enable complex organisation and retrieval of qualitative data. Framework analysis will be used as an established method of data analysis designed to gain insight and enhance understanding of social phenomena in order to influence social policy in the UK. The method has five distinct phases that are interlinked and form a methodical and rigorous framework to enable researchers to understand and interpret data, and move from descriptive accounts to a conceptual explanation of what is happening from the data of participants in the study. The method is transparent and enables teams of researchers to work together.

Concepts, categories and themes are identified and coded. All data relevant to each category will be compared with other data to provide analytical categories. As new categories emerge the process will be repeated and new questions will be formulated as a result. Exceptions to the generated theories will be sought to challenge any emerging findings. Interviewer bias will be minimised using the following method. Following discussion of emergent themes from the first five interviews in each category group, another researcher in the team will analyse the next five transcripts separately and the resulting thematic analysis will be compared with the primary researcher's codes. Categories will be developed in distinct phases that are interlinked and form a methodical and rigorous framework to enable researchers to understand and interpret data, and move from descriptive accounts to a conceptual explanation of what is happening from the data of participants in the study. The method is transparent and enables teams of researchers to work together.

10. Data management guidelines

All information collected will be kept strictly confidential. Any information which would allow individual participants or healthcare professionals to be identified will not be released. The project will comply with all aspects of the Data Protection Act 1998. Participant confidentiality and anonymity will be maintained throughout the duration of the project and in the dissemination of results.

Specific consent for how data will be stored, used and destroyed is sought using the participant Information Sheet and Consent Form. Personal details from women applying to participate in the NOSH Scheme will be maintained to allow the NOSH Office to administer the NOSH SCHEME. These data will include name, address, contact telephone numbers and/or email address, birth date of baby.

All participant data will be stored confidentially and securely in locked filing cabinets or on the Project Drive on University of Sheffield computers (accessible only to project personnel using password protected access) at ScHARR and will only be available to identified NOSH Project staff.

11. Trial monitoring and oversight

There will be no formal monitoring of the HCPs delivery of the NOSH Scheme

12. Adverse events

No adverse events have been identified in the feasibility stage to date. Risks including sending 6 month questionnaires to women whose baby has died, the administration of the questionnaires by health visitors who know the women should mean this risk is minimal.

A possibility has also been raised that vulnerable women will feel coerced into breastfeeding through the offer of a financial incentive. Ongoing qualitative work in the feasibility stage with both women and healthcare professionals (midwives and health visitors) has found no evidence of this to date. We will continue to be alert to this possibility during the trial.

13. Risks to delivery of the intervention

13.1 Extensive work with local collaborators

The extensive work conducted in the development and feasibility stages of the project with local stakeholders (and formal approvals already obtained) and ongoing key stakeholder involvement in the NOSH project will help minimise any risks associated with the guardians (healthcare providers) for each cluster. The risk that we foresee is outlined in the following section.

13.2 Continuation sites

The intervention was initially offered for three months in three clusters in the sampling frame (Sheffield City, and the towns of Chesterfield and Rotherham), the intervention is still being delivered in these three sites. We plan to continue these three sites to run alongside the trial for two reasons.
Firstly, withdrawal of the intervention from the feasibility sites at a time when other sites are being selected to be offered the intervention would reduce the good will towards the NOSH project from HCPs specialising in supporting infant feeding in these areas. Secondly, the 'continuation sites' will enable the project to gather valuable information on the longer term impact of the intervention and help build a pool of expertise in relation to the intervention amongst 'infant feeding' healthcare professionals.
The cost of this will be the cost of the vouchers (406 births in all three sites = £24,522) as the administration of these three smaller 'continuation' clusters can run alongside that of the trial clusters.

13.3 Risks to HV involvement in the trial

With the commissioning of health visiting being transferred from NHS England to public health in local authorities in 2015 (current date given is October 2015), we anticipated that this could impact on Health Visitor involvement in the delivery of the intervention in Sheffield in the very last stage of the trial. However, having consulted with the NHS England commissioners of the Health Visiting service for the South Yorkshire region we are reassured it is unlikely that Health Visitors will be asked to stop participating in the NOSH trial. In the unlikely event that HVs will have to stop participating then the NOSH research team will ask the midwives to co-sign and/or employ a private health visitor (see section 8.6. for the national situation).

14. Trial closure

14.11 End of trial

The trial will end on the last day when the NOSH Voucher claim form is accepted. This will be one month after when the youngest eligible baby is 6 months old (this will be seven months after the end of the birth eligibility period – see Figure 8.2). At this point the Declaration of End of Trial Form will be submitted to the participating ethical committee as required.

14.2 Archiving of trial documentation

At the end of the project, data will be securely archived at ScHARR at the University of Sheffield for 5 years, after which all data will be confidentially destroyed. If a participant withdraws consent for their data to be used it will be confidentially destroyed immediately.

14.33 Early discontinuation of trial

The trial may be stopped before completion by the Trial Steering Committee. All areas will be informed in writing of the reasons for early closure and the actions to be taken with regard to the delivery of the intervention.

15. Trial management and Trial committees

The NOSH project team are a multi-disciplinary team with extensive relevant academic expertise (clinical/ public health trial management, health economics, statistics and qualitative research) and clinical/ public health expertise (midwifery, health visitors, paediatrics, public health consultants). The Principal Investigator has overall responsibility for the design, co-ordination and management of the project.

15.1 Project Management Group responsible for ongoing management of the project which includes the trial

Name	Position
Dr Clare Relton	PI, Research Fellow, ScHARR
Dr Mark Strong	Clinical Senior Lecturer in Public Health, Public Health doctor and statistician
Dr Julia Burrows	Consultant in Public Health
Professor Kate Thomas	Honorary Professor Health Services Research
Dr Helen Baston	Consultant Midwife (Public Health), Sheffield Teaching Hospitals
Professor Mary Renfrew	Professor of Mother and Infant Health, University of Dundee
Dr Heather Whitford	Lecturer in Midwifery, University of Dundee
Elaine Scott	Project Manager
Professor Liddy Goyder	Deputy Dean of ScHARR and Professor of Public Health
Professor Julia Fox-Rushby	Professor of Health Economics, Brunel University
Dr Nana Anokye	Senior Research Fellow, HERG, Brunel University

15.2 Trial Steering Committee provides overall supervision of the trial and monitors trialprogress and conduct and advises on scientific credibility.

Name	Position	Independent
Dr Andrew Furber	Director of Public Health, Wakefield<br>(Chair)	Y
Professor Andrew Briggs	Professor of Health Economics and Health Technology Assessment, Glasgow University	Y
Professor David Tappin	Professor of Clinical Trials for Children, School of Medicine, University of Glasgow	Y
Dr Clare Relton	Principle Investigator (NOSH), Senior Research Fellow, ScHARR University of Sheffield	N
Prof Mary Renfrew	(Co-investigator) Professor of Mother and Infant Health, University of Dundee	N
Gavin Malloch	MRC Observer	Y
Prof Jon Nicholl	Representative of host institution<br>Dean of ScHARR, Professor of Health Services Research, University of Sheffield. Director NIHR School for Public Health Research	N
**Other observers as appropriate**		

Prof Kate Thomas	Honorary Professor of Health Services Research, ScHARR University of Sheffield	N
Professor Stephen Walters	Professor of Medical Statistics, ScHARR University of Sheffield	N
Elaine Scott	Project Manager of the NOSH Project	N

The final committee composition will be decided in discussion with the MRC/ NPRI committee.

16. Ethical and regulatory compliance

The trial will be conducted subject to Research Ethics Committee favourable opinion including any provisions for site specific assessment. The application will be re-submitted through the IRAS central allocation system. The approval letter from the ethics committee and copy of approved patient information leaflet, consent forms and any ethically approved questionnaires will be present in the site files before initiation of the study and patient recruitment. Local research governance approvals will be sought from all participating research sites.
This clinical trial will be conducted in accordance with The Medicines for Human Use (Clinical Trials) Regulations 2004 SI: 1031 plus subsequent amendments.

17. Sponsorship and indemnity

The trial has been financed by the NPRI (Phase IV Awards) and details have been drawn up in a separate agreement. This is a University of Sheffield sponsored study. The University of Sheffield provides insurance for this study. The University has in place insurance against liabilities for which it may be legally liable and this cover includes any such liabilities arising out of the NOSH research project/study

18. Publication policy

As this is an innovative randomised controlled trial it will be of interest to a wide variety of stakeholders. These will include policy makers such as the Department of Health, funders and providers of infant feeding services within the NHS and Public Health services within local authorities, to charitable bodies involved with infant feeding such as UNICEF, and to potential researchers and research funding bodies.
The findings of this research will be available to the NPRI, MRC, the public and other interested bodies. It will also be offered for presentation at clinical meetings and will be offered for publication in peer reviewed medical journals.

19. Abbreviations

BF	Breastfeeding
BFSWs	Breastfeeding support workers
CI	Confidence interval
DH	Department of Health
GI	Gastrointestinal
HCPs	Healthcare professionals
HVs	Health Visitors
ICC	Intra class correlation coefficient
ISRCTN	International Standard Randomised Controlled Trial Number
LRTI	Lower respiratory tract infection
MRC	Medical Research Council
MWs	Midwives
NIHR CRN	National Institute for Health Research Clinical Research Network
NOSH	Nourishing Start for Health
NPRI	National Prevention Research Initiative
p.a.	per annum
PSSRU	Personal Social Services Research Unit
QALY	Quality adjusted life year
RCT	Randomised controlled trial
REC	Research ethics committee
Wk	week
URMS	University Research Monitoring System

20. References

Caro JJ, Briggs AH, Siebert U, et al. 2012. Modeling good research practices – overview: A report of the ISPOR-SMDM modeling good research practices task force-1. *Value Health* 2012; 15:796-803

Carr et al. 2011. An evidence synthesis of qualitative and quantitative research on component intervention techniques, effectiveness, cost-effectiveness, equity and acceptability of different versions of health-related lifestyle advisor role in improving health. *Health Technol Assess* 2011 Feb; 15(9): iii-iv, 1-284

Department of Health. 2012. NHS Reference costs (2011/12). http://www.gov.uk/government/publications/nhs-reference-costs-financial-year-2011-to-2012

Department of Health. 2013. BF statistics: England. https://www.gov.uk/government/collections/breastfeeding-quarterly-statistics-england

Gomes et al. 2012a Methods for covariate adjustment in cost-effectiveness analysis that use cluster randomised trials**.** *Health Econ* 21(9): 1101-18.

Gomes et al. 2012b Developing appropriate methods for cost-effectiveness analysis of cluster randomized trials. *Med Decis Making* 32(2): 350-61

Gomes et al. 2012c. Statistical methods for cost-effectiveness analyses that use data from cluster randomized trials: a systematic review and checklist for critical appraisal. *Med Decis Making* 2012 Jan-Feb; 32(1): 209-20

Gomes et al 2013 Multiple imputation methods for handling missing data in cost-effectiveness analyses that use data from hierarchical studies: an application to cluster randomized trials. *Med Decis Making* 33(8): 1051-63

Groleau D et al. 2013. Power to negotiate spatial barriers to breastfeeding in a western context: when motherhood meets poverty. *Health and Place* 24 (2013): 250-259

Hawe P, Shiell A, Riley T, Gold L 2004. Methods for exploring implementation variation and local context within a cluster rendomised community intervention trial. *Journal of Epidemiology and Community Health*; 58(9): 788-93

Hoddinott P, Britten J, Pill R (2010). Why do interventions work in some places and not others: a breastfeeding support group trial. *Social Science and Medicine*; 70(5): 769-78

Jacklin P et al. 2007. Modelling the cost effectiveness of interventions to promote breastfeeding. NICE Maternal and Child Nutrition Programme, 2007, London

National Institute for Health and Care Excellence (NICE). 2013. Guide to the methods of technology appraisal. http://www.nice.org.uk/media/D45/1E/GuideToMethodsTechnologyAppraisal2013.pdf

NHS England. 2014. Health Visiting Programme. http://www.england.nhs.uk/ourwork/qual-clin-lead/hlth-vistg-prog/

Oakley A, Strange V, Bonell C, Allen E, Stephenson J, RIPPLE Study Team 2006. Process evaluation in randomised controlled trials of complex interventions. *British Medical Journal*; 332: 413-6

Relton C et al. 2013a. Behaviour change in breastfeeding: are financial incentives acceptable? Invited presentation BABCP (British Association for Behavioural Change and Cognitive Psychotherapies) conference July 2013, Imperial College London

Relton C et al. 2013b. Are financial incentives for breastfeeding acceptable? Poster presentation at the National Prevention Research Initiative (NPRI) conference December 2013, Oxford

Renfrew M et al. 2012. Preventing disease and saving resources: the potential contribution of increasing breastfeeding rates in the UK. UNICEF, 2012, London

Stapleton H et al. 2002. Qualitative study of evidence based leaflets in maternity care. *BMJ* 324: 1-6

Suwantika and Postma. 2013. Effect of breastfeeding promotion interventions on cost-effectiveness of rotavirus immunization in Indonesia. *BMC Public Health* 2013, 13: 1106

Whelan B et al. 2013a. Views on the acceptability of financial incentives for breastfeeding: a qualitative study. Public Health Science conference November 2013, Institute of Child Health, London

Whelan B et al. 2013b. Practical considerations in developing a financial incentive scheme for breastfeeding: a qualitative study of stakeholders' views. Presentation at the National Prevention Research Initiative (NPRI) conference December 2013, Oxford

Whelan B et al. 2014. Healthcare providers' views on the acceptability of financial incentives for breastfeeding: a qualitative study. BMC Pregnancy and Childbirth. 14:355 http://www.biomedcentral.com/1471-2393/14/355/abstract

Whitford H et al. 2014. A financial incentive scheme to encourage breastfeeding (NOSH) in low-income areas: designing the scheme incorporating key stakeholders' views. Submitted to *The Practicing Midwife*.

Willan AR, Briggs AH. 2006. Statistical analysis of cost-effectiveness data. *Pharmaceutical Statistics*; 9(4): 339-340

Appendix 1. Quebec ‘Nursing benefit’ Scheme

e for the Social Assistance Program or the Social Solidarity Program

Proper nutrition at birth = A healthy future adult!

SPECIAL
NURSING
BENEFIT

If you are breast-feeding a baby under 1 year of age, you are eligible for a special nursing benefit of $55 per month. This benefit will be paid every month until your baby turns 1 year of age, provided you continue breast-feeding during that time.

How to apply

File an application with your CLE that includes proof of your baby’s birth and an indication of how long you plan to breast-feed. It is important that you notify us if you stop breast-feeding.

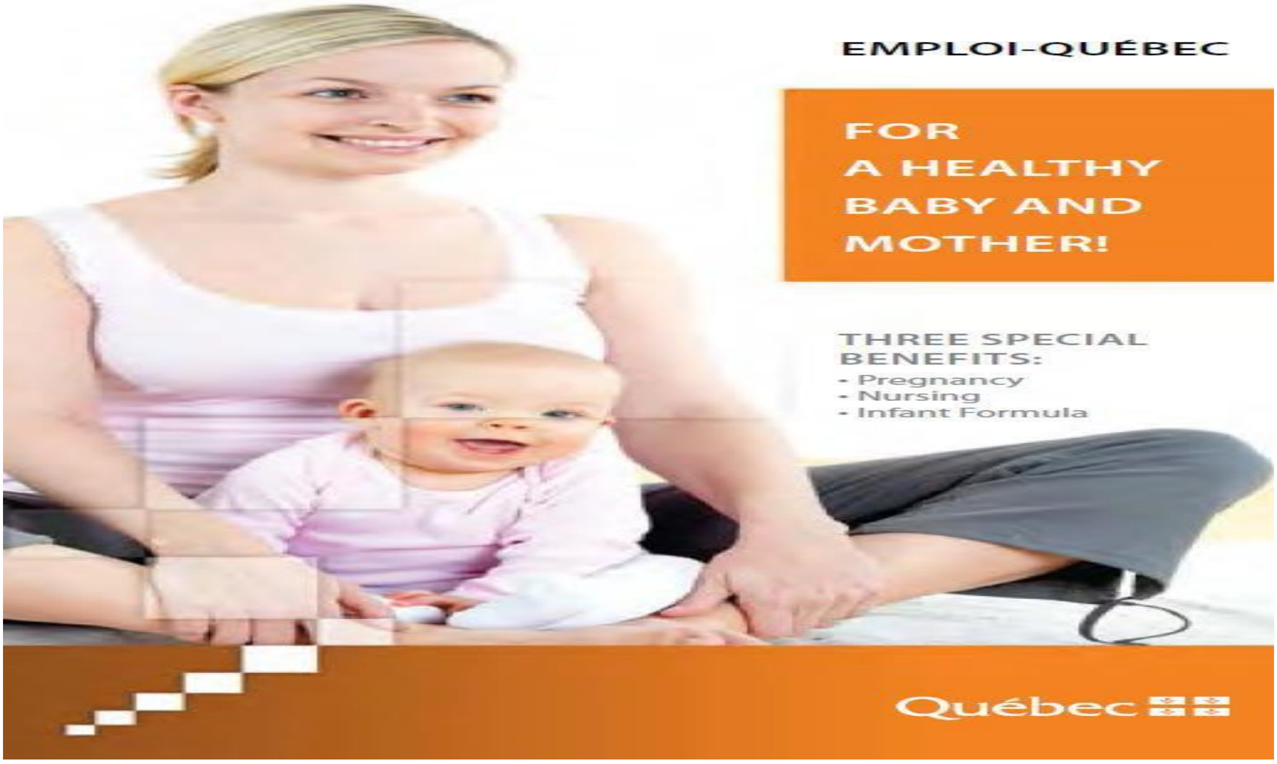

Appendix 2

NHS England Health Visiting Service Specification 2014/15
http://www.england.nhs.uk/wp-content/uploads/2014/03/hv-serv-spec.pdf.

This spec can be found in the resources section on the NHS England webpage. http://www.england.nhs.uk/ourwork/qual-clin-lead/hlth-vistg-prog/).

			Formula: Numerator / Denominator x 100		
Key Outcomes	Breastfeeding	Percentage of infants for whom breastfeeding status is recorded at 6-8wk check	Numerator: Number of infants where feeding status has been recorded at 6-8wk check<br>Denominator: Total number of infants due 6-8wk check<br>Formula: Numerator / Denominator x 100	*% prevalence target	Central collection (quarterly) Unify2
		Percentage of infants being breastfed at 6-8wks	Numerator: Number of infants recorded as being totally and partially breastfed at 6-8wks<br>Denominator: Total number of infants due 6-8wk check<br>Formula: Numerator / Denominator x 100	*% prevalence target	Central collection (quarterly) Unify2

Appendix 3. NOSH Booklet (sample)

About NOSH

NOSH is here to help you give your baby the most NOurishing Start for Health.

If you are giving your baby breast milk (breastfeeding or expressed breast milk) then you could get vouchers worth up to £200 for you and your baby.

Breast milk is perfectly designed for your baby and provides everything they need for around the first six months of their life. It helps to keep them healthy and protected from infections. What's more, it's free, ready prepared and always comes at the right temperature!

Contents

What do I get? 1
What are the vouchers for? 2
Do I qualify 3
How do I claim my vouchers? 4
When can I claim my vouchers? 5
What are the benefits of breastfeeding? 6
Where can I get support for breastfeeding? 7
Questions and Answers 8

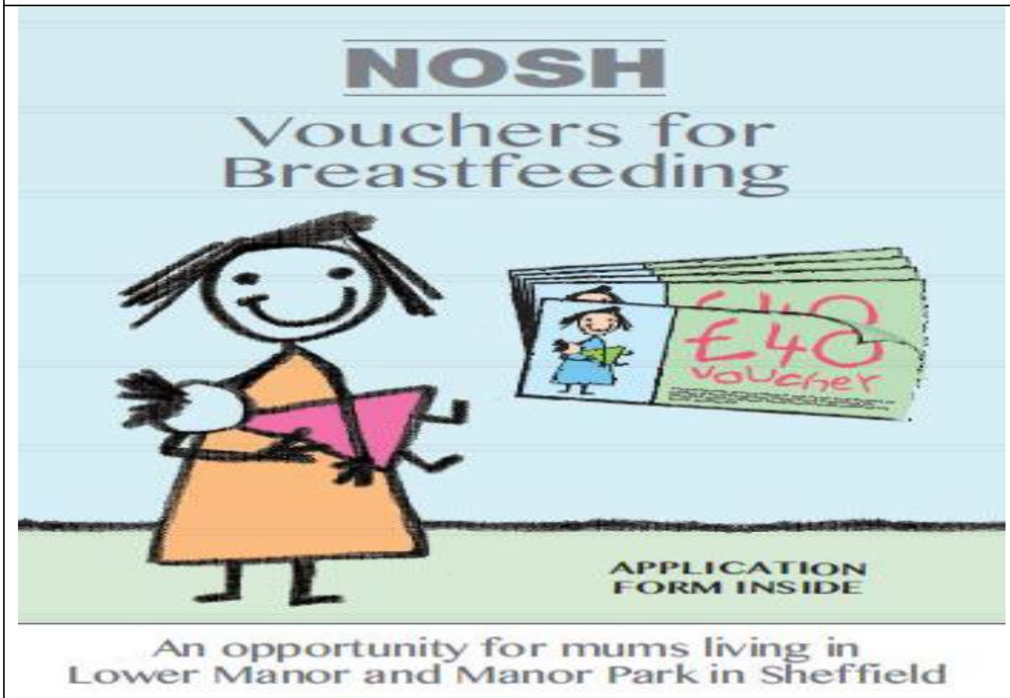

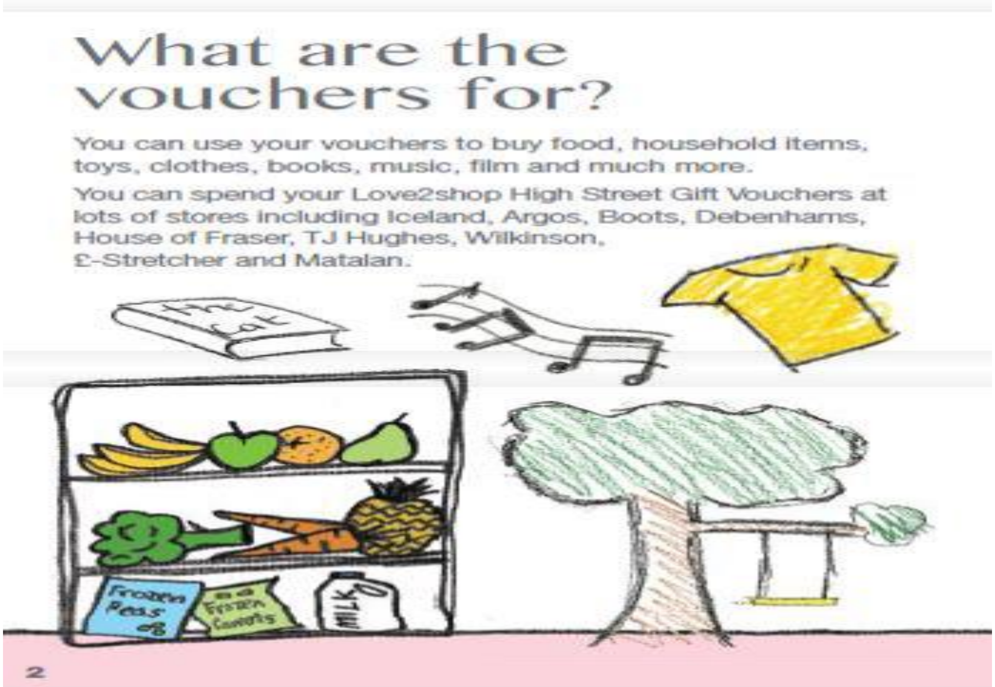
What are the vouchers for?
You can use your vouchers to buy food, household items, toys, clothes, books, music, film and much more.
You can spend your Love2shop High Street Gift Vouchers at lots of stores including Iceland, Argos, Boots, Debenhams, House of Fraser, TJ Hughes, Wilkinson, £-Stretcher and Matalan.
Frozen Peas
MILK
2

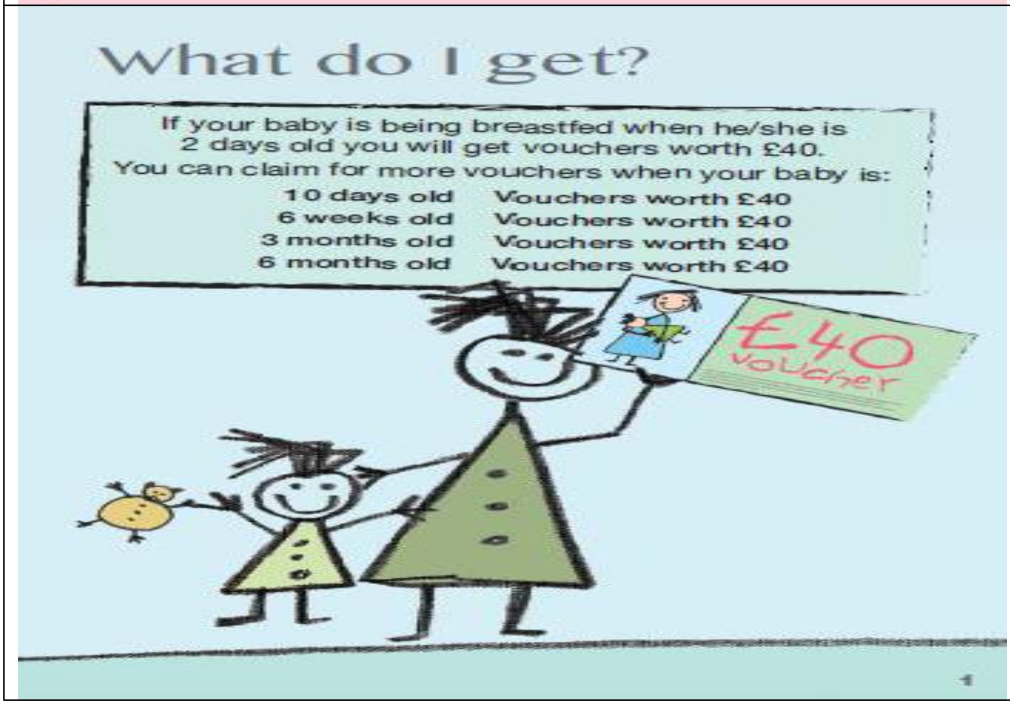
What do I get?
If your baby is being breastfed when he/she is 2 days old you will get vouchers worth £40.
You can claim for more vouchers when your baby is:
10 days old Vouchers worth £40
6 weeks old Vouchers worth £40
3 months old Vouchers worth £40
6 months old Vouchers worth £40
£40 voucher

How do I claim my vouchers?

STEP 1 Fill in the Application Form and ask your MIDWIFE or HEALTH VISITOR to sign it. Then send it to the **NOSH** Office. Use the FREEPOST envelope. We will then send you your CLAIM FORMS.

STEP 2 When you start giving your baby breastmilk, fill in the CLAIM FORM, ask your MIDWIFE or HEALTH VISITOR to sign it and send it back to us and we will send you your Love2shop vouchers by return post.

STEP 3 Spend your vouchers!

Our contact details

Still have some questions or want another copy of the application form? Please contact us at:

NOSH office: **0300 123 0188**

or email us at: **noshoffice@sheffield.ac.uk**

www.noshvoucher.org

4

Do I qualify?

You qualify for **NOSH** if you

- Live in **Lower Manor** or **Manor Park** in Sheffield. (Ring us on 0330 123 0188 or email noshoffice@sheffield.ac.uk to find out if you qualify)
- Have a baby born between November 1st 2013 and March 1st 2014
- Are aged 16 or over
- Are breastfeeding your baby or giving expressed breast milk

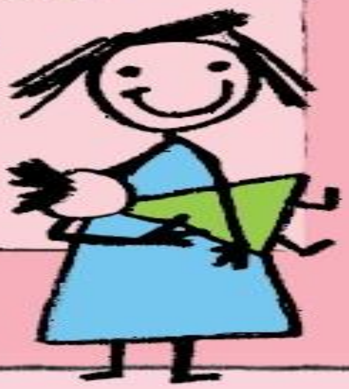

3

What are the benefits of breastfeeding?

What happens in your baby's first years has a big effect on how healthy he or she will be in the future. Breast milk gives your baby all the nutrients he or she needs for around the first 6 months of life and is beneficial beyond 6 months too. It helps to protect your baby from infection and other diseases, and also benefits you by reducing your chances of getting some illnesses later in life. All of these benefits can help save the NHS millions of pounds every year.

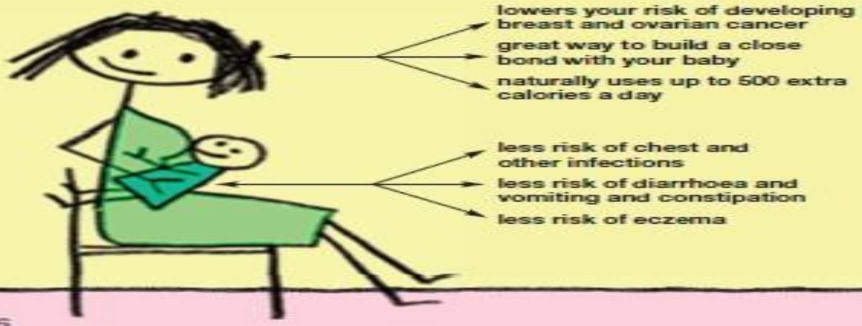

6

When can I claim my vouchers?

You can claim vouchers if you're breastfeeding when your baby is:

- ✓ 2 days old
- ✓ 10 days old
- ✓ 6 weeks old
- ✓ 3 months old
- ✓ 6 months old

We will send you £40 worth of vouchers for each claim you make.

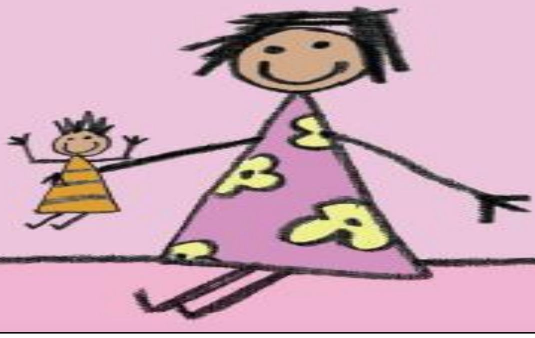

5

Questions and Answers

Q How will I get my vouchers?
A Your vouchers (worth £40) will be sent to you using Royal Mail. You will need to sign for them.

Q When will I get my vouchers?
A We will send you your vouchers as soon as we get your signed Claim Form.

Q How many vouchers will I get?
A You will get 8 x £5 vouchers (total value £40) for each signed completed Claim Form that you send us.

Q How many times can I claim NOSH Vouchers for Breastfeeding?
A If you breastfeed your baby until they are at least 6 - 8 weeks old then you can claim 3 times and receive vouchers worth a total of £120.
If you breastfeed your baby until they are 6 months old then you can claim 5 times and will receive vouchers worth a total of £200.

Q Can I still claim my vouchers if I occasionally give my baby infant formula?
A Yes. As long as you mainly give your baby breast milk you can claim your vouchers.

8

Where can I get support for breastfeeding?

Remember, when you are breastfeeding you are never alone. There are lots of places where you can get advice, information and help with breastfeeding. Ask your midwife or health visitor about breastfeeding support in your area, or contact your local children's centre. You can also talk to your local breastfeeding support worker at your children's centre (Breastfeeding support workers are mums who breastfed their own babies and are on hand to give you the help you need).

Your local contacts are:

Community midwives:
Midwifery Team: **07747 630951**

Health Visiting team

Childrens Centre

Manor Community Childcare Centre: 0114 264 2594

www.facebook.com/BreastfeedinginSheffield

There are also national charities that provide advice on breastfeeding, such as the Breastfeeding Network:
www.breastfeedingnetwork.org.uk
or National Breastfeeding Helpline: **0300 100 0212**

7

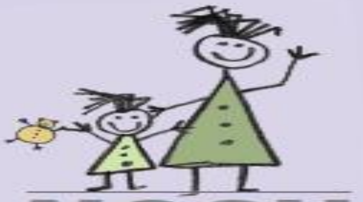

NOSH

Tel 0330 123 0188
noshoffice@sheffield.ac.uk
www.noshvoucher.org

*The **NOSH** Scheme offers vouchers to mums who breastfeed as a way of acknowledging both the value of breastfeeding to babies, mums and society, and the effort involved in breastfeeding.*

*The **NOSH** Scheme is being tested by researchers at the University of Sheffield (in collaboration with the universities of Dundee and Brunel), so it is only available in a few areas in the UK for a limited time.*

This research is fully funded by the National Prevention Research Initiative http://www.mrc.ac.uk/Ourresearch/ResearchInitiatives/NPRI/index.htm#P89_7386

The University Of Sheffield.

School of Health & Related Research (Public Health section)
University of Sheffield, Regent Court, 30 Regent Street, Sheffield, S1 4DA

Q What if I move house when I am on the scheme?

A Contact the **NOSH** office on noshoffice@sheffield.ac.uk with your new contact details.

Q My friend wants to breastfeed her baby and claim the NOSH Vouchers but she does not live in my area. Can she take part?

A The **NOSH** Vouchers for Breastfeeding Scheme is only being run in a few areas. Ask your friend to contact the **NOSH** Office and we will check whether she is eligible.

Q My baby is due after March 1st 2014 - can I still get vouchers for breastfeeding?

A Sorry, no. At the moment the **NOSH** Scheme is temporary while it is being tested.

Q I have lost my Claim Forms or FREEPOST envelope

A Contact the **NOSH** Office and we will send you some more.

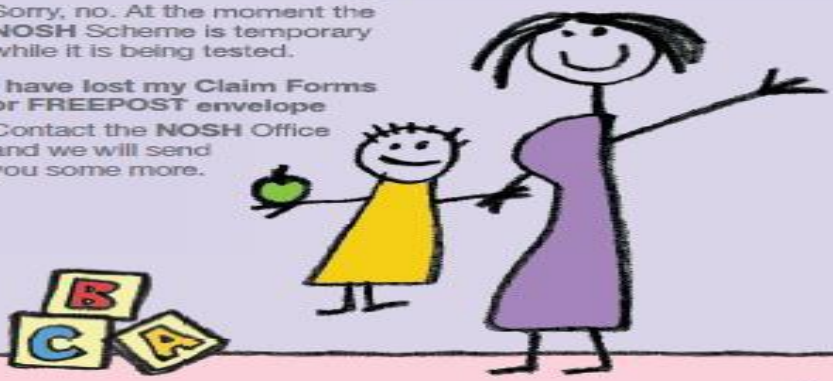

9

Appendix 4 Protocol version history

Protocol version/name	Body submitted to	Reference	Date of Submission	Outcome
Case for support/original bid (NOSH Protocol v0.1)	MRC/National Prevention Research Initiative Phase 4	G1002475	10/5/2011	Successful; grant awarded 22/2/12
NOSH DEVELOPMENT STAGE 1st June 2012 to 30th May 2013 Full research protocol March 2013				
NOSH 'Women's Views' short protocol	School of Health and Related Research Ethics Committee (ScHARR REC)	0591/KW	10/7/12	Approved 19/7/13
First amendment	ScHARR REC	0591/KW	3/9/12	Approved 18/9/12
Second amendment	ScHARR REC	0591/KW	1/10/12	Approved 2/10/12
Third amendment and 'Street Survey' short protocol v1.0	ScHARR REC	0591/KW	19/12/12	Approved 20/12/12
NOSH 'Professionals' Views' short protocol v1.2	National Institute for Health Research (NIHR) portfolio adoption	Study ID 13879	22/11/12	Included on NIHR CRN Portfolio on 14/12/12
	Sheffield City Council Research Governance Framework	N/A	4/12/12	Approved 6/12/12
	NHS R&D Sheffield Teaching Hospitals NHS FT	IRAS Ref: 117430	6/12/12	Project authorisation 31/1/13
	NHS R&D Sheffield Children's Hospital	IRAS Ref: 117430	6/12/12	Project authorisation 31/1/13
	NHS R&D Sheffield Health & Social Care Trust (Sheffield PCT)	IRAS Ref: 117430	6/12/12	Project authorisation 14/2/13
	Rotherham Metropolitan Borough Council and Young People's Services	N/A	11/2/13	Approved 11/6/13
	NHS R&D Rotherham NHS Foundation Trust	IRAS Ref: 117430	29/4/13	Project authorisation 21/5/13
NOSH FEASIBILITY STAGE 1st June 2013 to 30th May 2014				
NOSH Feasibility & Trial protocol v1.0	National Institute for Health Research portfolio adoption	Study ID 15385	25/4/13	NIHR CRN Portfolio on 30/10/13
NOSH Feasibility and cluster RCT Protocol	NRES West Midlands (Edgbaston) for Proportionate Review	Rec ref: 13/WM/0299	10/7/13	Favourable opinion 26/7/13
Minor amendment	NRES West Midlands	13/WM/0299	30/8/13	Approved 31/8/13
Minor amendment	NRES West Midlands	13/WM/0299	20/9/13	Approved 27/9/13
Minor amendment	NRES West Midlands	13/WM/0299	24/10/13	Approved 30/10/13
Substantial amendment	NRES West Midlands	13/WM/0299	19/3/14	Awaiting opinion
	NHS R&D Sheffield Teaching Hospitals NHS FTrust	IRAS Ref: 126464	1/8/13	Project authorisation 17/10/13
	NHS R&D Sheffield Children's Hospital	IRAS Ref: 126464	1/8/13	Project authorisation 16/9/13
	NHS R&D Rotherham NHS Foundation Trust	IRAS Ref: 126464	1/8/13	Project authorisation 11/12/13
	NHS R&D Chesterfield Royal NHS Foundation Trust	IRAS Ref: 126464	1/8/13	Project authorisation received 7/11/13
	NHS R&D Derbyshire Community Health Services NHS Foundation Trust	IRAS Ref: 126464	1/8/13	Project authorisation 30/10/13
	Sheffield City Council Research Governance Framework	N/A	23/7/13	Approved Research Governance Office 5/9/13, then rejected

				by Councillor 2/10/13
	Derbyshire County Council	N/A	24/7/13	Approved 21/10/13
	Rotherham Metropolitan Borough Council and Young People's Service	N/A	23/7/13	Approved 30/8/13
NOSH RCT PHASE 1st June 2014 to 30th May 2016				
NOSH Trial protocol V2	Medical Research Council		7/4/14	
NOSH Trial protocol V3	Medical Research Council		11/10/14	
